# Correction: Global Genetic Variations Predict Brain Response to Faces

**DOI:** 10.1371/journal.pgen.1004802

**Published:** 2014-10-14

**Authors:** The PLOS Genetics Staff

## Notice of Republication

This article was republished on September 12^th^ 2014, to correct errors in the order of the author list that were introduced during the typesetting process. Please download this article again to view the correct version. The originally published, uncorrected article and the republished, corrected article are provided here for reference.

## Supporting Information

File S1 Originally published, uncorrected article.
(PDF)

File S2 Republished corrected article.
(PDF)
