# Supplementary figures and images for: Global Genetic Variations Predict Brain Response to Faces
Source: PLoS Genet. 2014 Aug 14;10(8):e1004523. doi: 10.1371/journal.pgen.1004523 (PMC4133042; doi:10.1371/journal.pgen.1004523)

Ambiguous Faces

Angry Faces

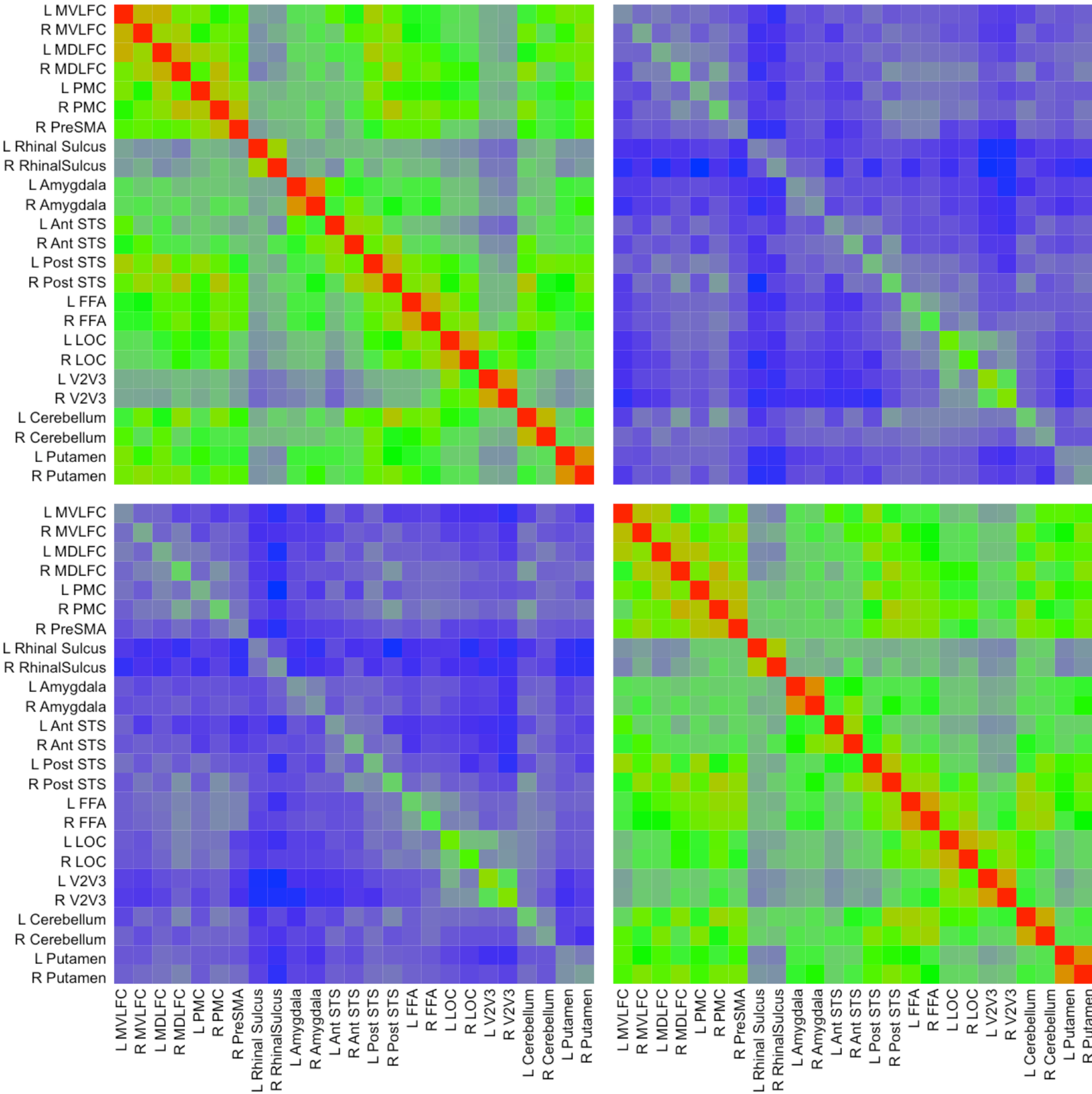

Ambiguous Faces

Angry Faces

Supplement: Figure S1 — Phenotypic (% BOLD Signal Change) correlation matrices for the Ambiguous and Angry contrasts. Mid-ventrolateral frontal cortex (MVLFC); Mid-dorsolateral frontal cortex (MDLFC); premotor cortex (PMC), pre supplementary motor area (PreSMA); superior temporal sulcus (STS); fusiform face area (FFA); lateral occipital cortex (LOC); left (L); right (R). (PDF) [file pgen.1004523.s001.pdf]

VG/Vp for %BSC

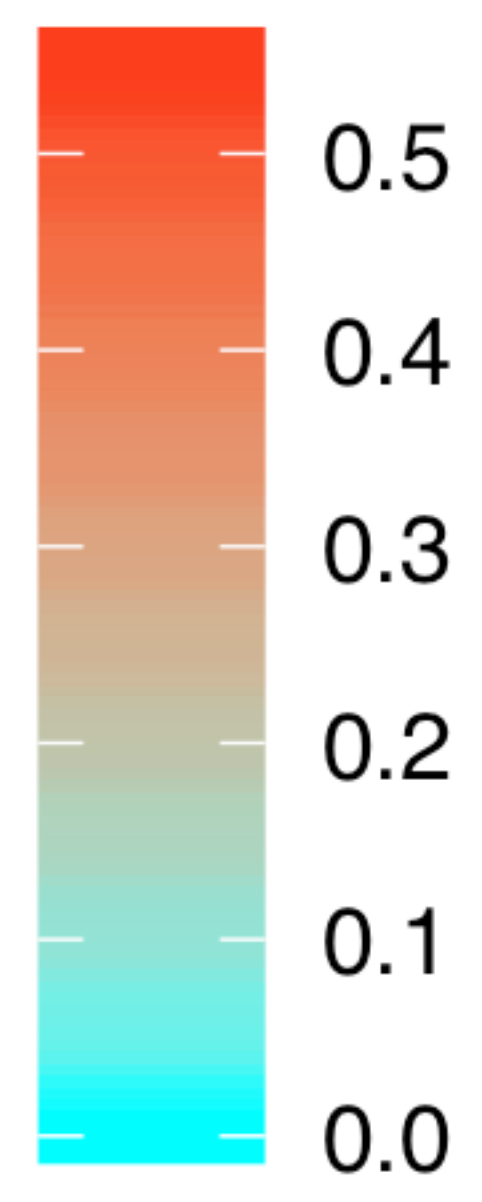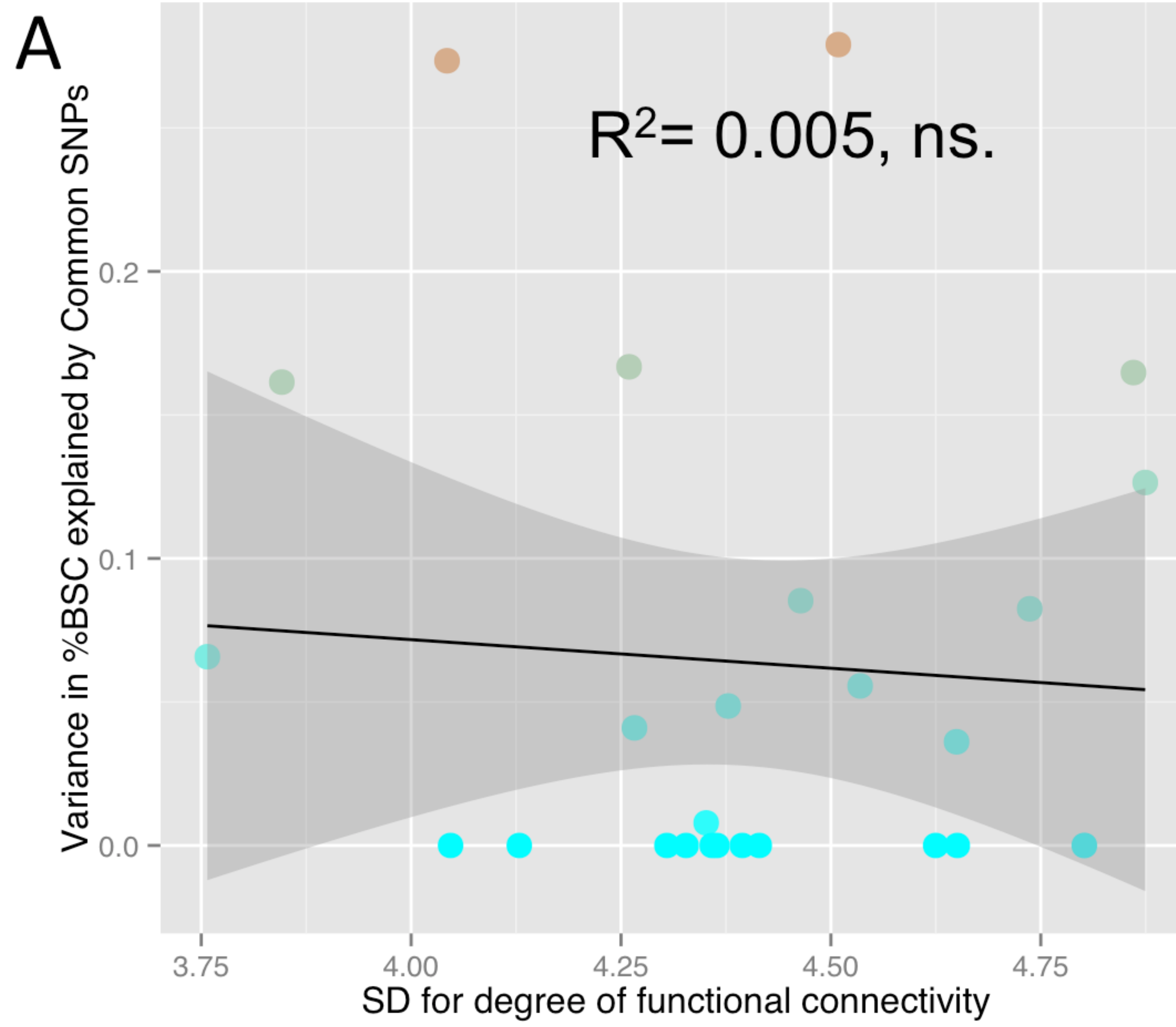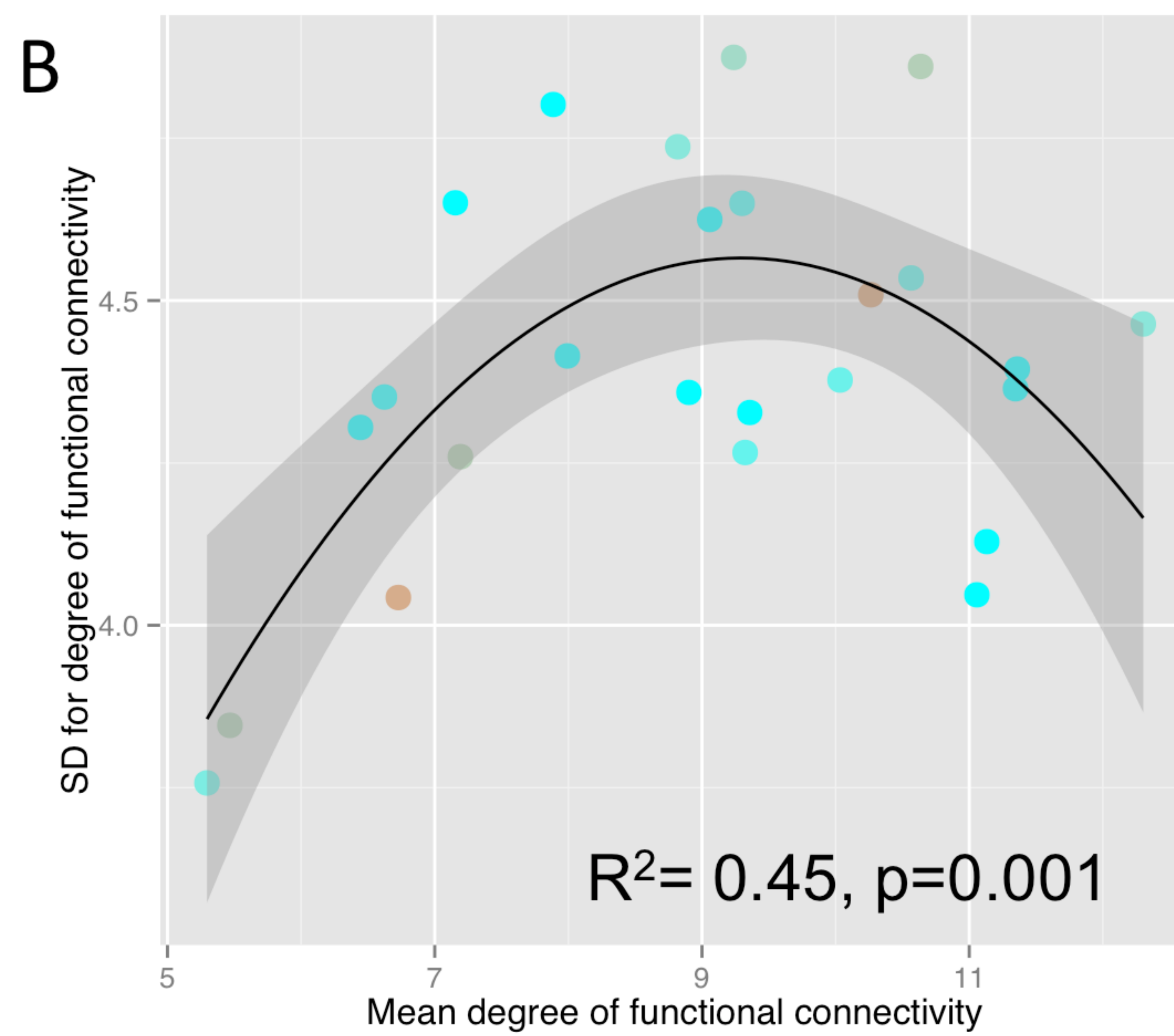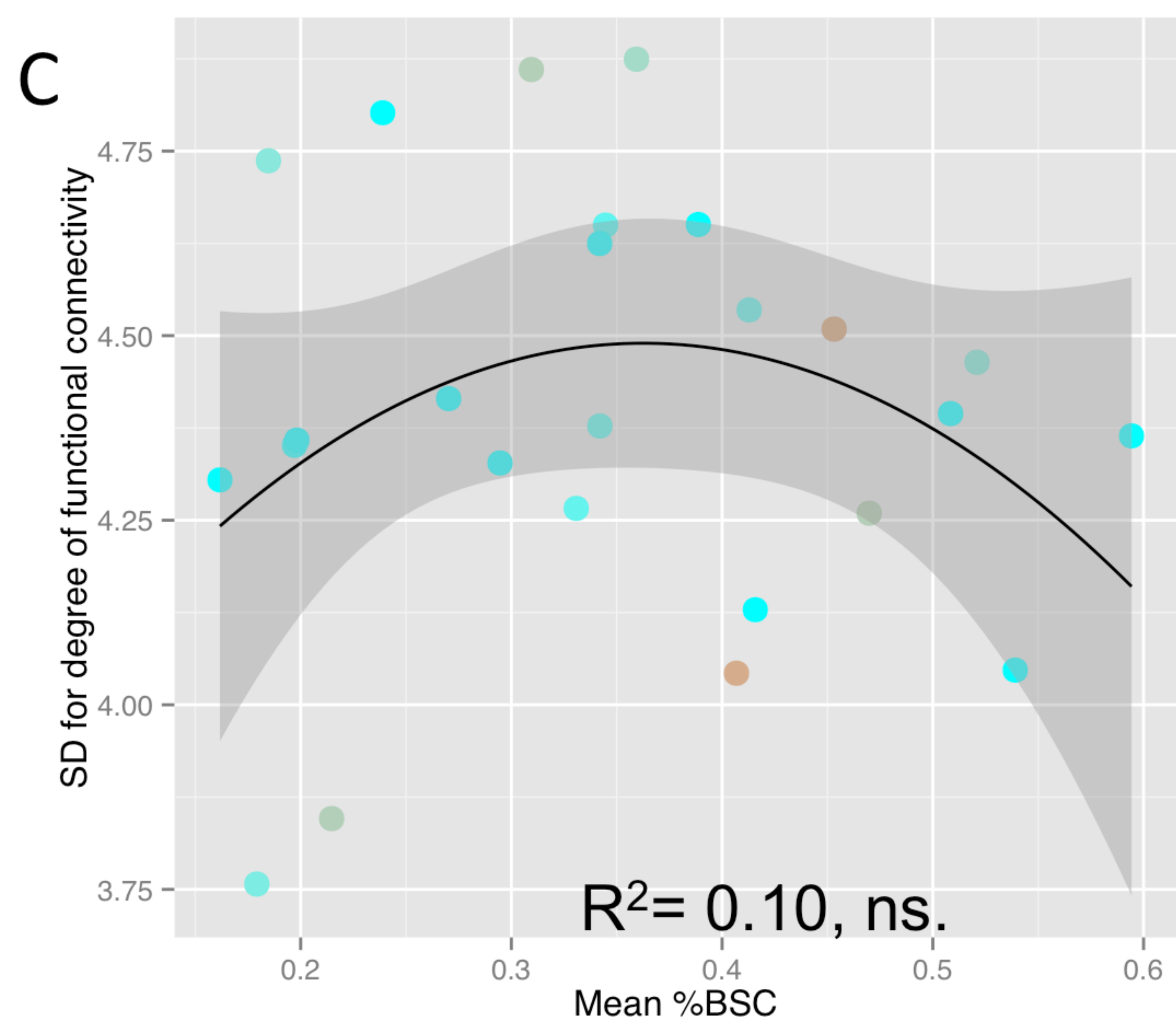

Supplement: Figure S2 — A) Relationship between GREML-based heritability estimates (Genetic Variance/Phenotypic Variance) and the population variance (standard deviation, SD) of functional connectivity (node degree) across 25 ROIs for the Angry face viewing contrast. B) Relationship between the population variance (SD) and the population mean of degree across the 25 ROIs. C) Relationship between population mean of the brain response (percent BOLD signal change, %BSC) and the population variance (SD) of degree across the 25 ROIs. For all three plots, colour is scaled according to the GREML results for %BSC (cyan for low values and red for high values). VG, Genetic Variance; Vp, Phenotypic Variance. (PDF) [file pgen.1004523.s002.pdf]

# Males

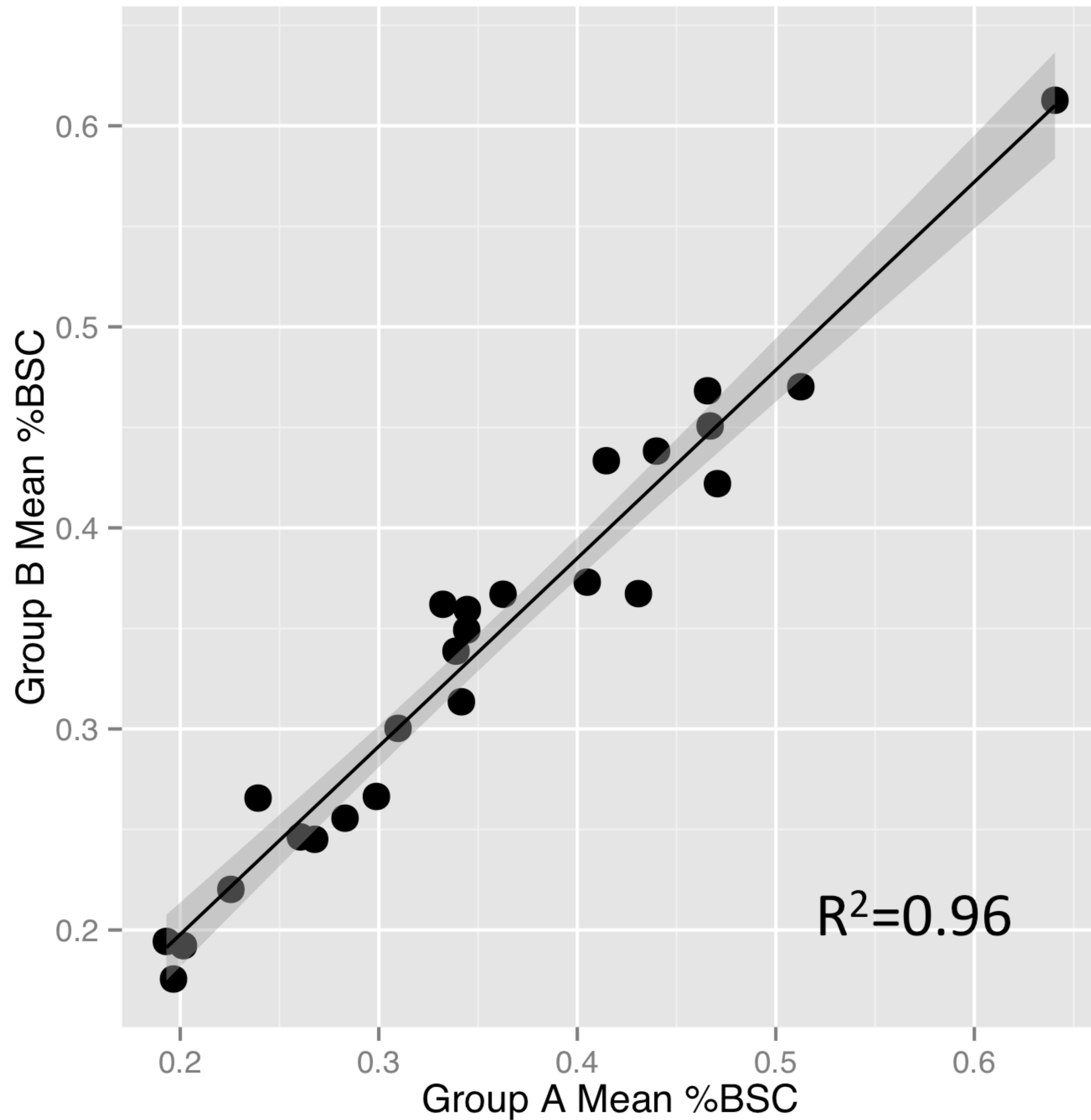

# Females

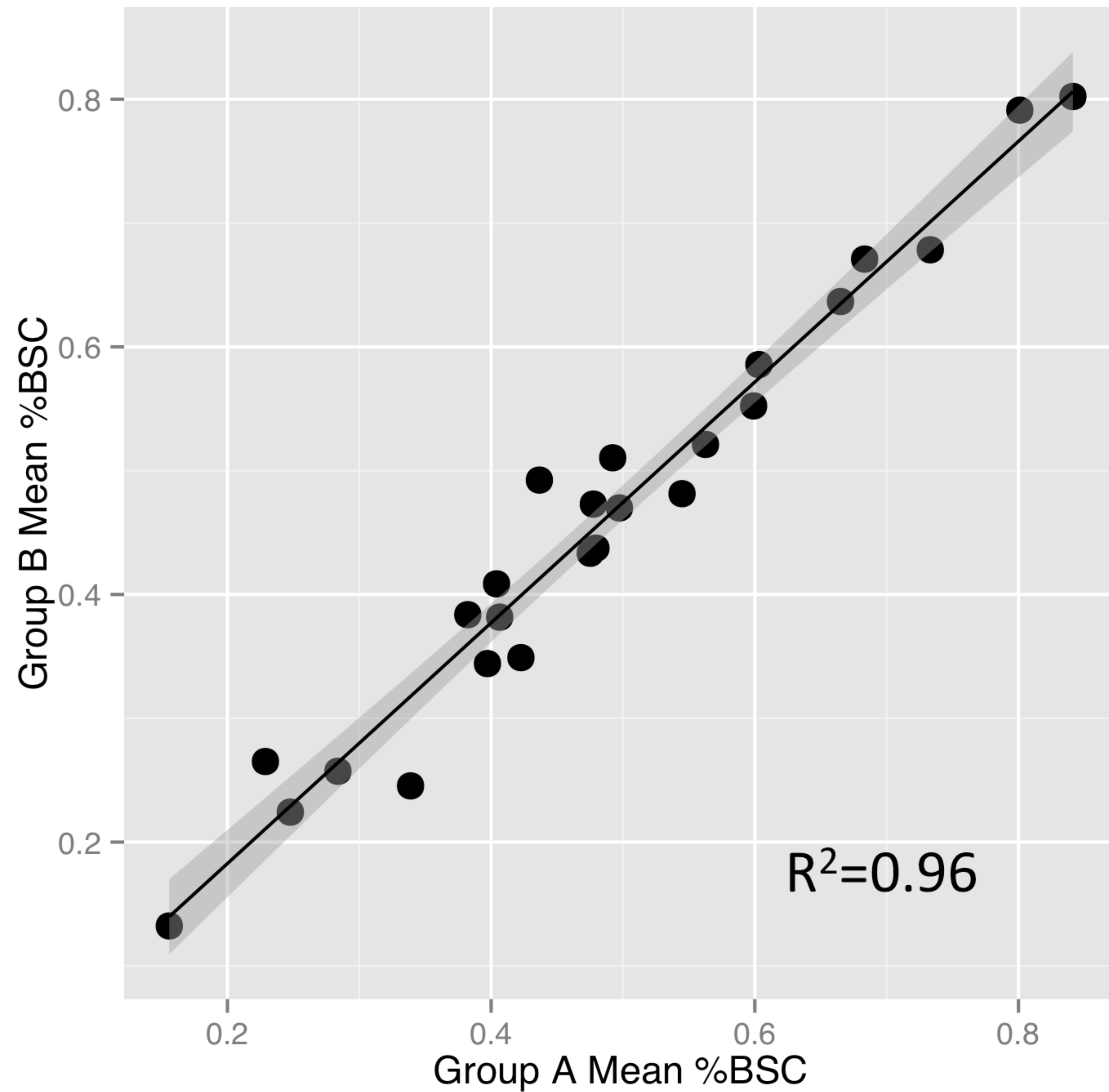

Supplement: Figure S3 — Brain response (% BOLD Signal Change) response across the 25 ROIs measured in Group A and B. Left, males (Group A, n = 434; Group B, n = 448); Right, females (Group A, n = 483; Group B, n = 459). (PDF) [file pgen.1004523.s003.pdf]
