# Supplementary material for: Global Genetic Variations Predict Brain Response to Faces
Source: PLoS Genet. 2014 Aug 14;10(8):e1004523. doi: 10.1371/journal.pgen.1004523 (PMC4133042; doi:10.1371/journal.pgen.1004523)
Supplement: Table S1 — GREML results for percent BOLD Signal Change (%BSC) in response to Ambiguous Facial expressions (vs. Control Stimuli) in 1,620 adolescents. Regions in bold are those with GREML-based estimates of heritability of the brain response significant at an alpha 0.05 (uncorrected). The critical value for X2(1) in this context is 2.7055. Mid-ventrolateral frontal cortex (MVLFC); Mid-dorsolateral frontal cortex (MDLFC); premotor cortex (PMC), pre supplementary motor area (PreSMA); superior temporal sulcus (STS); fusiform face area (FFA); lateral occipital cortex (LOC); left (L); right (R). VG, Genetic Variance; Vp, Phenotypic Variance; df, degrees of freedom. (DOC) [file pgen.1004523.s004.doc]

Supplemental Table S1: GREML results for percent BOLD Signal Change (%BSC) in response to Ambiguous Facial expressions (vs. Control Stimuli) in 1,620 adolescents.

Regions in bold are those with GREML-based estimates of heritability of the brain response significant at an alpha 0.05 (uncorrected). The critical value for ^2^(1) in this context is 2.7055.

| Region | VG | Vp | VG/Vp | VG/Vp Standard Error | Χ^2^ | df | p-value |
| --- | --- | --- | --- | --- | --- | --- | --- |
| L MVLFC | 0.287 | 0.997 | 0.288 | 0.220 | 1.750 | 1 | 0.090 |
| R MVLFC | 0.316 | 0.991 | 0.319 | 0.225 | 1.944 | 1 | 0.080 |
| L MDLFC | 0.260 | 0.972 | 0.267 | 0.220 | 1.504 | 1 | 0.100 |
| **R MDLFC** | **0.500** | **0.958** | **0.522** | **0.221** | **5.387** | **1** | **0.010** |
| **L PMC** | **0.367** | **0.983** | **0.373** | **0.220** | **2.909** | **1** | **0.040** |
| **R PMC** | **0.341** | **0.968** | **0.352** | **0.215** | **2.837** | **1** | **0.050** |
| **R PreSMA** | **0.467** | **0.986** | **0.474** | **0.214** | **5.165** | **1** | **0.010** |
| L Rhinal Sulcus | 0.000 | 0.996 | 0.000 | 0.227 | 0.000 | 1 | 0.500 |
| R RhinalSulcus | 0.310 | 1.005 | 0.309 | 0.223 | 1.900 | 1 | 0.080 |
| L Amygdala | 0.206 | 1.000 | 0.206 | 0.226 | 0.798 | 1 | 0.200 |
| R Amygdala | 0.306 | 1.002 | 0.305 | 0.224 | 1.822 | 1 | 0.090 |
| L Ant STS | 0.320 | 1.001 | 0.320 | 0.221 | 2.090 | 1 | 0.070 |
| **R Ant STS** | **0.541** | **1.003** | **0.540** | **0.225** | **5.409** | **1** | **0.010** |
| L Post STS | 0.285 | 0.981 | 0.291 | 0.219 | 1.811 | 1 | 0.090 |
| **R Post STS** | **0.371** | **0.963** | **0.385** | **0.221** | **3.043** | **1** | **0.040** |
| L FFA | 0.209 | 0.970 | 0.215 | 0.224 | 0.913 | 1 | 0.200 |
| R FFA | 0.167 | 0.959 | 0.174 | 0.220 | 0.640 | 1 | 0.200 |
| L LOC | 0.171 | 0.977 | 0.175 | 0.225 | 0.591 | 1 | 0.200 |
| R LOC | 0.183 | 0.964 | 0.190 | 0.218 | 0.790 | 1 | 0.200 |
| **L V2V3** | **0.545** | **1.006** | **0.541** | **0.218** | **6.143** | **1** | **0.007** |
| R V2V3 | 0.202 | 0.997 | 0.202 | 0.222 | 0.824 | 1 | 0.200 |
| L Cerebellum | 0.462 | 0.994 | 0.465 | 0.223 | 4.232 | 1 | 0.020 |
| **R Cerebellum** | **0.084** | **0.997** | **0.085** | **0.222** | **0.146** | **1** | **0.400** |
| L Putamen | 0.114 | 0.992 | 0.115 | 0.220 | 0.281 | 1 | 0.300 |
| **R Putamen** | **0.371** | **0.991** | **0.374** | **0.222** | **2.776** | **1** | **0.050** |

Mid-ventrolateral frontal cortex (MVLFC); Mid-dorsolateral frontal cortex (MDLFC); premotor cortex (PMC), pre supplementary motor area (PreSMA); superior temporal sulcus (STS); fusiform face area (FFA); lateral occipital cortex (LOC); left (L); right (R). VG, Genetic Variance; Vp, Phenotypic Variance; df, degrees of freedom.
