# Supplementary material for: Global Genetic Variations Predict Brain Response to Faces
Source: PLoS Genet. 2014 Aug 14;10(8):e1004523. doi: 10.1371/journal.pgen.1004523 (PMC4133042; doi:10.1371/journal.pgen.1004523)
Supplement: Table S2 — GREML results for percent BOLD signal change (%BSC) in response to Angry Facial expressions (vs. Control Stimuli) in 1,620 adolescents. The critical value for X2(1) in this context is 2.7055. Mid-ventrolateral frontal cortex (MVLFC); Mid-dorsolateral frontal cortex (MDLFC); premotor cortex (PMC), pre supplementary motor area (PreSMA); superior temporal sulcus (STS); fusiform face area (FFA); lateral occipital cortex (LOC); left (L); right (R). VG, Genetic Variance; Vp, Phenotypic Variance; df, degrees of freedom. (DOC) [file pgen.1004523.s005.doc]

Supplemental Table S2: GREML results for percent BOLD signal change (%BSC) in response to Angry Facial expressions (vs. Control Stimuli) in 1,620 adolescents.

The critical value for Χ^2^(1) in this context is 2.7055.

Mid-ventrolateral frontal cortex (MVLFC); Mid-dorsolateral frontal cortex (MDLFC); premotor cortex (PMC), pre supplementary motor area (PreSMA); superior temporal sulcus (STS); fusiform face area (FFA); lateral occipital cortex (LOC); left (L); right (R). VG, Genetic Variance; Vp, Phenotypic Variance; df, degrees of freedom.

| Region | VG | Vp | VG/Vp | VG/Vp Standard Error | Χ^2^ | df | p-value |
| --- | --- | --- | --- | --- | --- | --- | --- |
| L MVLFC | 0.000 | 0.998 | 0.000 | 0.222 | 0.000 | 1 | 0.500 |
| R MVLFC | 0.036 | 0.998 | 0.036 | 0.220 | 0.028 | 1 | 0.400 |
| L MDLFC | 0.000 | 0.996 | 0.000 | 0.217 | 0.000 | 1 | 0.500 |
| R MDLFC | 0.279 | 1.000 | 0.279 | 0.226 | 1.471 | 1 | 0.100 |
| L PMC | 0.082 | 0.998 | 0.082 | 0.222 | 0.139 | 1 | 0.400 |
| R PMC | 0.055 | 0.995 | 0.056 | 0.223 | 0.061 | 1 | 0.400 |
| R PreSMA | 0.000 | 0.991 | 0.000 | 0.221 | 0.000 | 1 | 0.500 |
| L Rhinal Sulcus | 0.065 | 0.992 | 0.066 | 0.223 | 0.087 | 1 | 0.400 |
| R RhinalSulcus | 0.161 | 0.997 | 0.161 | 0.223 | 0.519 | 1 | 0.200 |
| L Amygdala | 0.274 | 1.001 | 0.273 | 0.215 | 1.712 | 1 | 0.100 |
| R Amygdala | 0.167 | 0.999 | 0.167 | 0.217 | 0.615 | 1 | 0.200 |
| L Ant STS | 0.008 | 0.987 | 0.008 | 0.219 | 0.001 | 1 | 0.500 |
| R Ant STS | 0.126 | 0.997 | 0.126 | 0.227 | 0.296 | 1 | 0.300 |
| L Post STS | 0.165 | 1.000 | 0.165 | 0.223 | 0.545 | 1 | 0.200 |
| R Post STS | 0.085 | 0.994 | 0.085 | 0.220 | 0.154 | 1 | 0.300 |
| L FFA | 0.000 | 0.985 | 0.000 | 0.221 | 0.000 | 1 | 0.500 |
| R FFA | 0.000 | 0.986 | 0.000 | 0.215 | 0.000 | 1 | 0.500 |
| L LOC | 0.000 | 0.996 | 0.000 | 0.219 | 0.000 | 1 | 0.500 |
| R LOC | 0.000 | 0.992 | 0.000 | 0.219 | 0.000 | 1 | 0.500 |
| L V2V3 | 0.041 | 0.999 | 0.041 | 0.223 | 0.033 | 1 | 0.400 |
| R V2V3 | 0.000 | 0.998 | 0.000 | 0.218 | 0.000 | 1 | 0.500 |
| L Cerebellum | 0.049 | 1.000 | 0.049 | 0.218 | 0.051 | 1 | 0.400 |
| R Cerebellum | 0.000 | 0.994 | 0.000 | 0.216 | 0.000 | 1 | 0.500 |
| L Putamen | 0.000 | 0.983 | 0.000 | 0.218 | 0.000 | 1 | 0.500 |
| R Putamen | 0.000 | 0.997 | 0.000 | 0.221 | 0.000 | 1 | 0.500 |
