# Supplementary material for: Global Genetic Variations Predict Brain Response to Faces
Source: PLoS Genet. 2014 Aug 14;10(8):e1004523. doi: 10.1371/journal.pgen.1004523 (PMC4133042; doi:10.1371/journal.pgen.1004523)
Supplement: Table S3 — Population mean (Mean) and population variance (Standard deviation, SD) for Percent BOLD Signal Change (Angry Faces vs. Control Stimuli) and the Degree of Functional Connectivity (number of regions correlated with an r>0.3). Mid-ventrolateral frontal cortex (MVLFC); Mid-dorsolateral frontal cortex (MDLFC); premotor cortex (PMC), pre supplementary motor area (PreSMA); superior temporal sulcus (STS); fusiform face area (FFA); lateral occipital cortex (LOC); left (L); right (R). (DOC) [file pgen.1004523.s006.doc]

Supplemental Table S3: Population mean (Mean) and population variance (Standard deviation, SD) for Percent BOLD Signal Change (Angry Faces vs. Control Stimuli) and the Degree of Functional Connectivity (number of regions correlated with an r>0.3).

Mid-ventrolateral frontal cortex (MVLFC); Mid-dorsolateral frontal cortex (MDLFC); premotor cortex (PMC), pre supplementary motor area (PreSMA); superior temporal sulcus (STS); fusiform face area (FFA); lateral occipital cortex (LOC); left (L); right (R).

| Region of Interest | Percent BOLD Signal Change | |  | Degree of Functional Connectivity | |
| --- | --- | --- | --- | --- | --- |
|  | Mean | Variance |  | Mean | Variance |
| L MVLFC | 0.27 | 0.81 |  | 7.99 | 4.41 |
| R MVLFC | 0.34 | 0.72 |  | 9.30 | 4.65 |
| L MDLFC | 0.34 | 0.69 |  | 9.06 | 4.62 |
| R MDLFC | 0.45 | 0.62 |  | 10.26 | 4.51 |
| L PMC | 0.18 | 0.66 |  | 8.82 | 4.74 |
| R PMC | 0.41 | 0.65 |  | 10.56 | 4.53 |
| R PreSMA | 0.39 | 0.84 |  | 7.15 | 4.65 |
| L Rhinal Sulcus | 0.18 | 0.50 |  | 5.30 | 3.76 |
| R RhinalSulcus | 0.21 | 0.40 |  | 5.47 | 3.85 |
| L Amygdala | 0.41 | 0.66 |  | 6.73 | 4.04 |
| R Amygdala | 0.47 | 0.62 |  | 7.19 | 4.26 |
| L Ant STS | 0.20 | 0.70 |  | 6.62 | 4.35 |
| R Ant STS | 0.36 | 0.55 |  | 9.24 | 4.87 |
| L Post STS | 0.31 | 0.56 |  | 10.64 | 4.86 |
| R Post STS | 0.52 | 0.53 |  | 12.30 | 4.46 |
| L FFA | 0.51 | 0.66 |  | 11.36 | 4.39 |
| R FFA | 0.59 | 0.74 |  | 11.35 | 4.36 |
| L LOC | 0.42 | 0.71 |  | 11.13 | 4.13 |
| R LOC | 0.54 | 0.72 |  | 11.06 | 4.05 |
| L V2V3 | 0.33 | 0.58 |  | 9.32 | 4.27 |
| R V2V3 | 0.29 | 0.58 |  | 9.36 | 4.33 |
| L Cerebellum | 0.34 | 0.57 |  | 10.03 | 4.38 |
| R Cerebellum | 0.20 | 0.66 |  | 8.90 | 4.36 |
| L Putamen | 0.16 | 0.62 |  | 6.44 | 4.30 |
| R Putamen | 0.24 | 0.60 |  | 7.89 | 4.80 |
