# Supplementary material for: Global Genetic Variations Predict Brain Response to Faces
Source: PLoS Genet. 2014 Aug 14;10(8):e1004523. doi: 10.1371/journal.pgen.1004523 (PMC4133042; doi:10.1371/journal.pgen.1004523)
Supplement: Table S4 — Results of bivariate analysis of genetic covariances in percent BOLD Signal Change (%BSC) in response to Ambiguous Facial expressions (vs. Control Stimuli) in 1,620 adolescents across regions of interest (ROI) constituting the “Optional” MVLFCR, MDLFCL, PMCL, AntSTSR) and “Obligatory” (PostSTSR, FFAR, LOCL, LOCR) Networks. Regions in bold and underlined are those with GREML-based estimates of genetic covariance (rG) of the brain response in a given pair of ROIs significant at an alpha 0.05 and 0.1 (uncorrected). Standard errors of the estimates are in parentheses. Mid-ventrolateral frontal cortex (MVLFC); Mid-dorsolateral frontal cortex (MDLFC); premotor cortex (PMC), superior temporal sulcus (STS); fusiform face area (FFA); lateral occipital cortex (LOC); left (L); right (R); Ant, Anterior; Post, posterior. (DOC) [file pgen.1004523.s007.doc]

Supplemental Table S4. Results of bivariate analysis of genetic covariances in percent BOLD Signal Change (%BSC) in response to Ambiguous Facial expressions (vs. Control Stimuli) in 1,620 adolescents across regions of interest (ROI) constituting the “Optional” R MVLFC, L MDLFC, L PMC, R AntSTS) and “Obligatory” (R PostSTS, R FFA, L LOC, R LOC) Networks.

Regions in bold and underlined are those with GREML-based estimates of genetic covariance (rG) of the brain response in a given pair of ROIs significant at an alpha 0.05 and 0.1 (uncorrected). Standard errors of the estimates are in parentheses.

Mid-ventrolateral frontal cortex (MVLFC); Mid-dorsolateral frontal cortex (MDLFC); premotor cortex (PMC), superior temporal sulcus (STS); fusiform face area (FFA); lateral occipital cortex (LOC); left (L); right (R); Ant, Anterior; Post, posterior.

|  | R MVLFC | L MDLFC | L PMC | R AntSTS | R PostSTS | R FFA | L LOC | R LOC |
| --- | --- | --- | --- | --- | --- | --- | --- | --- |
| R MVLFC | --- |  |  |  |  |  |  |  |
| L MDLFC | 0.87 (0.26*)* | ------ |  |  |  |  |  |  |
| L PMC | 0.62 (0.31) | 0.86 (0.23) | ------ |  |  |  |  |  |
| R Ant STS | **1.00 (0.26)** | 0.64 (0.30) | 0.58 (0.27) | ------ |  |  |  |  |
| R Post STS | **0.88 (0.22)** | 0.56 (0.32) | 0.63 (0.28) | **0.93 (0.16)** | ------ |  |  |  |
| R FFA | 1.0 (0.58) | 1.0 (0.49) | 0.67 (0.41) | 0.28 (0.45) | 0.78 (0.32) | ------ |  |  |
| L LOC | 0.47 (0.53) | 0.80 (0.49) | 0.45 (0.47) | 0.40 (0.44) | 0.81 (0.43) | 0.44 (0.69) | ------ |  |
| R LOC | 0.13 (0.64) | 0.20 (0.65) | 0.48 (0.44) | 0.20 (0.46) | 0.39 (0.45) | 0.13 (0.86) | 0.90 (0.31) | ------ |
