# Supplementary material for: Correction: Global Genetic Variations Predict Brain Response to Faces
Source: PLoS Genet. 2014 Oct 14;10(10):e1004802. doi: 10.1371/journal.pgen.1004802 (PMC4197032; doi:10.1371/journal.pgen.1004802)
Supplement: File S1 — Originally published, uncorrected article. (PDF) [file pgen.1004802.s001.pdf]

PLOS GENETICS

Global Genetic Variations Predict Brain Response to Faces

Erin W. Dickie[1], Amir Tahmasebi[1,2], Leon French[1], Natasa Kovacevic[1], Tobias Banaschewski[3,4], Gareth J. Barker[5], Arun Bokde[6], Christian Büchel[7], Patricia Conrod[5,8], Herta Flor[3,4], Hugh Garavan[6,9], Juergen Gallinat[10], Penny Gowland[11], Andreas Heinz[10], Bernd Ittermann[12], Claire Lawrence[13], Karl Mann[3,4], Jean-Luc Martinot[14], Frauke Nees[3,4], Thomas Nichols[15], Mark Lathrop[16], Eva Loth[5,17], Zdenka Pausova[18], Marcela Rietschel[3,4], Michal N. Smolka[19,20], Andreas Ströhle[10], Roberto Toro[21], Gunter Schumann[5,17], Tomáš Paus[1,13]* and the IMAGEN consortium¶

1 Rotman Research Institute, University of Toronto, Toronto, Ontario, Canada, **2** Philips Research North America, Briarcliff Manor, New York, United States of America, **3** Central Institute of Mental Health, Mannheim, Germany, **4** Medical Faculty Mannheim, University of Heidelberg, Heidelberg, Germany, **5** Institute of Psychiatry, King's College London, London, United Kingdom, **6** Institute of Neuroscience, Trinity College Dublin, Dublin, Ireland, **7** Universitaetsklinikum Hamburg Eppendorf, Hamburg, Germany, **8** Department of Psychiatry, Universite de Montreal, CHU Ste Justine Hospital, Montreal, Quebec, Canada, **9** Departments of Psychiatry and Psychology, University of Vermont, Burlington, Vermont, United States of America, **10** Department of Psychiatry and Psychotherapy, Charité – Universitätsmedizin Berlin, Berlin, Germany, **11** Sir Peter Mansfield MR Centre, University of Nottingham, Nottingham, United Kindom, **12** Physikalisch-Technische Bundesanstalt (PTB), Braunschweig und Berlin, Berlin, Germany, **13** School of Psychology, University of Nottingham, Nottingham, United Kindom, **14** Institut National de la Santé et de la Recherche Médicale, INSERM CEA Unit 1000 ''Imaging & Psychiatry'', University Paris Sud, Orsay, and AP-HP Department of Adolescent Psychopathology and Medicine, Maison de Solenn, University Paris Descartes, Paris, France, **15** University of Warwick, Coventry, United Kingdom, **16** Centre National de Génotypage, Evry, France, **17** MRC Social, Genetic and Developmental Psychiatry (SGDP) Centre, London, United Kingdom, **18** The Hospital for Sick Children, University of Toronto, Toronto, Ontario, Canada, **19** Department of Psychiatry and Psychotherapy, Technische Universität Dresden, Dresden, Germany, **20** Neuroimaging Center, Department of Psychology, Technische Universität Dresden, Dresden, Germany, **21** Institute Pasteur, Paris, France

Abstract

Face expressions are a rich source of social signals. Here we estimated the proportion of phenotypic variance in the brain response to facial expressions explained by common genetic variance captured by ~500,000 single nucleotide polymorphisms. Using genomic-relationship-matrix restricted maximum likelihood (GREML), we related this global genetic variance to that in the brain response to facial expressions, as assessed with functional magnetic resonance imaging (fMRI) in a community-based sample of adolescents (n = 1,620). Brain response to facial expressions was measured in 25 regions constituting a face network, as defined previously. In 9 out of these 25 regions, common genetic variance explained a significant proportion of phenotypic variance (40–50%) in their response to ambiguous facial expressions; this was not the case for angry facial expressions. Across the network, the strength of the genotype-phenotype relationship varied as a function of the inter-individual variability in the number of functional connections possessed by a given region ($R^2 = 0.38$, $p < 0.001$). Furthermore, this variability showed an inverted U relationship with both the number of observed connections ($R^2 = 0.48$, $p < 0.001$) and the magnitude of brain response ($R^2 = 0.32$, $p < 0.001$). Thus, a significant proportion of the brain response to facial expressions is predicted by common genetic variance in a subset of regions constituting the face network. These regions show the highest inter-individual variability in the number of connections with other network nodes, suggesting that the genetic model captures variations across the adolescent brains in co-opting these regions into the face network.

Citation: Dickie EW, Tahmasebi A, French L, Kovacevic N, Banaschewski T, et al. (2014) Global Genetic Variations Predict Brain Response to Faces. PLoS Genet 10(8): e1004523. doi:10.1371/journal.pgen.1004523

Editor: Peter M. Visscher, The University of Queensland, Australia

Received December 6, 2013; **Accepted** May 30, 2014; **Published** August 14, 2014

Copyright: © 2014 Dickie et al. This is an open-access article distributed under the terms of the Creative Commons Attribution License, which permits unrestricted use, distribution, and reproduction in any medium, provided the original author and source are credited.

Funding: IMAGEN receives research funding from the European Community's Sixth Framework Programme (LSHM-CT-2007-037286). Further support was provided by the UK Department of Health NIHR-Biomedical Research Centre ''Mental Health'' and the MRC programme grant ''Developmental pathways into adolescent substance abuse'' (93558). The funders had no role in study design, data collection and analysis, decision to publish, or preparation of the manuscript.

Competing Interests: The authors have declared that no competing interests exist.

* Email: tpaus@research.baycrest.org

¶ Membership of the IMAGEN consortium is provided in the Acknowledgments.

Introduction

Interactions with peers are of high relevance to our mental health. Patients with various psychological disorders show impairments in face perception and emotion recognition [1–4]. Similarly, differential neural responses to faces have been reported in various psychological disorders including depression [5], psychopathy and/or aggressive tendencies [6], autism [7], and schizophrenia [8].

Our ability to process faces is modulated by both environment and genes. Using a twin design, Zhu and colleagues observed that inter-individual variations in face perception are heritable, with

Author Summary

We measured brain response to facial expressions in a large sample of typically developing adolescents (n = 1,620) and assessed "heritability" of the response using common genetic variations across the genome. In a subset of brain regions, we explained 40–50% of phenotypic variance by genetic variance. These brain regions appear to differ from the rest of the face network in the degree of inter-individual variations in their functional connectivity. We propose that these regions, including the prefrontal and premotor cortex, represent "Optional" part of the network co-opted by its "Obligatory" members, including the posterior part of the superior temporal sulcus, fusiform face area and the lateral occipital cortex, concerned with processing complex visual stimuli.

the genetic component as high as 50% in adolescents performing various face tasks [9]. Although the key elements of the neural network underlying face processing are well known [10], whether or not brain response to facial expressions show comparable levels of heritability is unknown.

Here we address this question using genomic-relationship-matrix restricted maximum likelihood (GREML), applied using Genome-wide Complex Trait Analysis (GCTA) software [11] to a dataset of functional magnetic resonance images (fMRI) obtained in over 1,600 typically developing adolescents while they were observing videoclips of ambiguous or angry facial expressions [The IMAGEN Study; 12]. The GREML approach allows one to estimate how much phenotypic variance is attributable to the genetic variance captured by *all* common genetic variations (single nucleotide polymorphisms; SNPs) assayed in a typical Genome Wide Association Study (GWAS). We will ask here whether "heritability" of the response to facial expressions – as estimated using the GREML approach – varies across these regions. We will then examine possible reasons for such regional variations.

Results

The GREML-based metrics were calculated from a total of 1,620 unrelated adolescents (age M(SD) = 14.4(0.39) range 12.7–16.3 years, n = 879 male, n = 945 female) with complete, quality controlled fMRI and genomic data (511,089 SNPs).

In fMRI, brain response to a stimulus is inferred from the variations in hemodynamics detected as the blood oxygenation-level dependent (BOLD) signal on T_2*-weighted MR images. This signal relies on the fact that brain activity is associated with an oversupply of oxygenated blood to the brain region engaged by the stimulus; consequently, small veins that drain this region contain some of the unused oxygenated blood. Thus, the BOLD signal reflects the proportion of oxygenated and de-oxygenated blood in a given brain region at a given moment; most likely, this hemodynamic signal is proportional to the local field potentials generated by (excitatory) inputs.

Here, summary measures for the BOLD response (%BSC) were calculated for two face viewing conditions (Ambiguous movements and Angry Expressions), each compared with a non-biological motion control condition; this was done for 25 brain regions of interest (ROIs), as defined previously using a probabilistic map of the brain response to facial expressions [13]. GREML-based estimates of "heritability" were calculated using the GCTA package [13][11]. We observed significant estimates of "heritability" for the Ambiguous vs. Control contrast %BSC in 9 out of these 25 ROIs (Fig. 1, Supplementary Table S1). No significant estimates were observed for any ROI in the Angry vs. Control contrast (Supplementary Table S2). By chance we would expect 25*0.05 = 1.25 false positives when examining 25 ROIs (in each contrast), or 2.5 when examining 50 ROIs (both contrasts combined) at the $\alpha = 0.05$ level. As we found 9 ROIs with $P < 0.05$ this appears to be evidence against the null hypothesis. To provide a P-value for this count-rate approach, we conducted a Monte Carlo simulation for each contrast using the observed correlation matrix (see Supplementary Figure S1 for the phenotypic correlation matrix between all 25 ROIs) [14]. Using 50,000 realizations, we simulated the null-hypothesis test statistics for each ROI (using correlated outcomes) and tabulated the distribution of the number of P-values significant at 0.05; this empirical distribution can be used to compute P-values for this count statistic. For the Ambiguous Facial Expressions contrast, this simulation confirmed that we could reject the null hypothesis of observing 9 significant tests by chance at P value of 0.03. For Angry Facial Expressions contrast, the P-value must be 1.0 for a count of 0 significant tests.

To illustrate the relationship between the GREML-based estimates of heritability (VG/Vp) and the number of SNPs with p values lower than a certain threshold, we have calculated Pearson's correlation coefficients between these two measures across 25 ROIs, as done by Yang and colleagues in their GREML-based study of 47 different traits [15]. We obtained the following results: $p<0.001$: $R^2 = 0.08$; $p<0.01$: $R^2 = 0.47$; $p<0.05$: $R^2 = 0.54$; $p<0.1$: $R^2 = 0.74$; $p<0.15$: $R^2 = 0.82$; $p<0.2$: $R^2 = 0.76$; $p<0.25$: $R^2 = 0.74$; and $p<0.3$: $R^2 = 0.64$. In Figure 2, we plot the number of SNPs with $p<0.15$ and the VG/Vp values across the 25 ROIs.

Given the high heritability of general intelligence [16], we have examined the possibility that intelligence correlates with the inter-individual variations in the brain response to facial expressions in the Ambiguous condition. In a subset of 1,772 individuals with available scores on four subtests of the Wechsler Intelligence Scale for Children – IV (similarities, vocabulary, block design and matrix reasoning), we found no correlation between these scores and the mean %BSC across all ROIs ($p>0.3$) or between the scores and the mean %BCS in the Optional ($p>0.2$) and Obligatory ($p>0.4$) networks. The lack of the relationship between general intelligence and %BSC suggests that the former does not contribute to the above heritability estimates of the brain response to the ambiguous facial expressions.

Next, we asked whether the above GREML-based estimates of heritability reflect any properties of the brain response across the examined ROIs. For example, are the ROIs with stronger response to facial expressions more heritable? Table 1 provides the population means and standard deviations for %BSC for all ROIs in the ambiguous contrast. Neither the population means ($r = -0.28$, $p = 0.18$) nor the population variance ($r = 0.07$, $p = 0.73$) of %BSC values across the 25 ROIs predicts the GREML-based (Genetic Variance [VG]/Phenotypic Variance [Vp]) estimates of heritability.

The brain regions considered here may be viewed as nodes of a "face" network. The strength of each region's contribution to this network may differ across regions (ROIs) and across individuals. To quantify this phenomenon, we extracted mean time-courses in the BOLD signal from all ROIs in each participant and used these to calculate matrices of functional connectivity for all participants. From these matrices, we estimated the number of connections of a given region with the other members of the face network using the graph-theory metric of nodal "degree" [17].

Table 1 provides the population means and standard deviations for the nodal degree for all ROIs (Ambiguous Facial expressions).

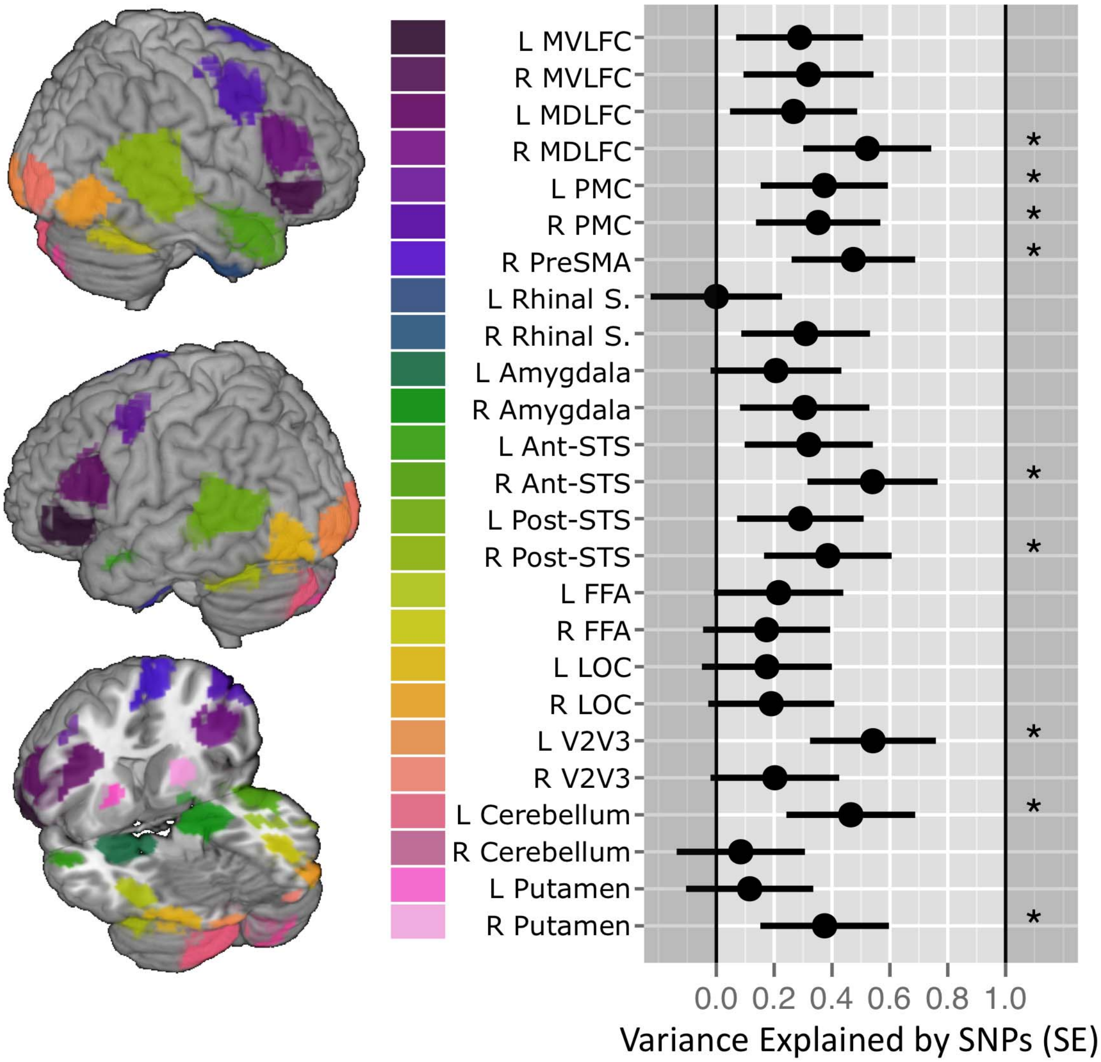

Figure 1. SNP-based estimates of heritability in the brain response to ambiguous faces. (Left) Locations of the 25 functional Regions of Interest (ROI) defined for the Dynamic Ambiguous Face vs. Control contrast [13]**.** (Right) Proportion of variance in mean percent BOLD signal change (%BCS) explained by common genetic variance for each ROI when viewing facial expressions in 1,620 unrelated adolescents. Error bars indicate the standard Error of the estimate. Stars indicate those estimates significant at an alpha 0.05 (uncorrected). Vertical gridlines are at intervals of 0.2. Abbreviations: Mid-ventrolateral frontal cortex (MVLFC); Mid-dorsolateral frontal cortex (MDLFC); premotor cortex (PMC), pre supplementary motor area (PreSMA); superior temporal sulcus (STS); fusiform face area (FFA); lateral occipital cortex (LOC); left (L); right (R). SNP, single nucleotide polymorphisms.
doi:10.1371/journal.pgen.1004523.g001

We then examined whether differences in this measure of functional connectivity across the 25 ROIs constituting the face network predict their GREML-based estimates of heritability (VG/Vp estimates). This is the case: the population (inter-individual) variance in the nodal degree predicts strongly the "heritability" ($R^2 = 0.38$, $p < 0.001$, Fig. 3a). Furthermore, the population variance in the nodal degree shows an inverse U-shaped relationship with the mean nodal degree (2nd order polynomial fit: $R^2 = 0.48$, $F(2,22) = 10.32$, $p < 0.001$, Fig. 3b) and the mean %BSC (2nd order polynomial fit: $R^2 = 0.32$, $F(2,22) = 5.07$ $p = 0.02$, Fig. 3c). Similarity of this inverse U-shaped relationship for the mean nodal degree and the mean %BSC is not surprising given a strong correlation between these two measures ($r = 0.84$, $p < 0.001$). Note, however, that the population means of nodal degree do not predict their GREML-based estimates of heritability across the 25 brain regions ($R^2 = 0.06$, $p = 0.23$). This is likely related to the fact that the mean nodal degree shows an inverted-U relationship with the population variance of this measure across these regions (Fig. 3b). Finally, we have repeated these analyses for the Angry Facial expressions; the only significant relationship observed in this condition was that between the population variance and the population mean in nodal degree (Supplementary Figure S2). Population means and standard deviations for %BSC and nodal degree for all ROIs of the Angry condition are given in Supplemental Table S3.

To illustrate the relationship between population variance and mean in the number of connections (nodal degree) in the Ambiguous contrast, we selected two groups of ROIs that differ in the combination of these two measures of degree: (1) ROIs with the highest variance and an intermediate mean; and (2) ROIs with the highest mean and the lowest variance. As shown in Figure 4 (and Table 2), proportion of individuals with connections between a given pair of ROIs (i.e., pair-wise correlations with $r > 0.3$) is intermediate

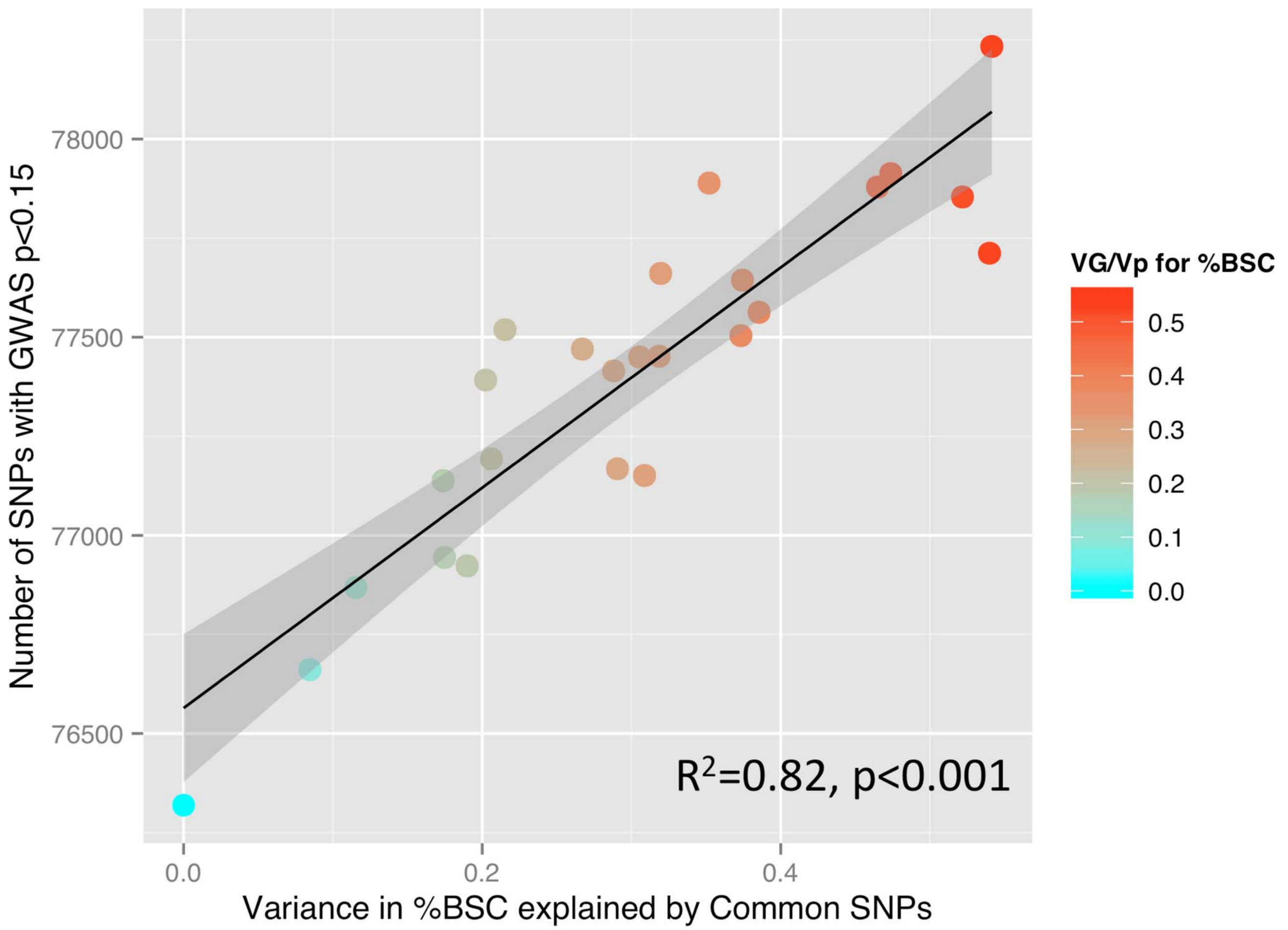

Figure 2. SNP-based estimates of heritability and the number of SNPs detected in a GWAS. Relationship between GREML-based heritability estimates (Genetic Variance/Phenotypic Variance) and the number of single nucleotide polymorphisms (SNPs) showing a significant relationship with percent BOLD signal change (%BSC) at a threshold of $p<0.15$ (see the Results section for rationale). P values for this analysis were obtained using linear regression.
doi:10.1371/journal.pgen.1004523.g002

within the first subnetwork (30 to 60% of participants) and very high within the second subnetwork (70 to 96% of participants). Importantly, the posterior STS (region #5 in Fig. 4) appears to act as a "bridge" between the two subnetworks: it is the only member of the second subnetwork with connections to all four nodes of the first subnetwork in 50% (or more) of participants. We then examined genetic covariance within and between the two subnetworks, which we term "Optional" and "Obligatory" (see Discussion for comparison with the "Extended" and "Core" systems of Haxby and colleagues [10]). Using a bivariate GCTA approach [18], we observed significant ($p<0.05$) genetic covariances in three pairs of ROIs: [R MVLFC - R Ant STS], [R MVLFC – R Post STS] and [R AntSTS – R Post STS]. Furthermore, we found marginal ($p<0.1$) covariances in four additional pairs of ROIs, three within the "Optional" network and one between the "Optional" and "Obligatory" network (L PMC - R Post STS). The full genetic-covariance matrix for the eight ROIs constituting the two subnetworks is provided in Supplemental Table S4.

Discussion

Using the GREML approach, we show that the aggregate of common genetic variations across the entire genome predicts brain response to facial expressions. This is the case, however, only for some brain regions when viewing ambiguous facial expressions. The fact that inter-individual variations in certain brain responses to certain faces can be predicted by genetic variations (across the entire genome) is in keeping with other evidence supporting heritability of many behavioral aspects of face perception. Indeed, prosopagnosia - an inability of recognize faces - is transmitted in some family pedigrees carrying an autosomal dominant pattern of transmission [19]. Using a twin design in adults, Wilmer et al. [20] reported that facial recognition has a strong heritable component. Greenwood et al. [21] found that memory for faces, as well as emotional recognition, were heritable phenotypes in a study of adult patients with schizophrenia and their non-affected relatives. Most relevant to the current study, Zhu et al. [9] showed that several aspects of face perception, including facial recognition and the face-inversion effect, were heritable and that the heritability increased from childhood to adolescence.

Heritability of the brain response to faces has been estimated in a couple of twin studies. Thus, Anokhin et al. [22] used event-related potentials to changes in emotional expression in children and reported heritability values varying between 42 and 64% [22]. Moreover, Polk et al. [23] showed that fMRI responses to faces in the ventral visual stream were more similar within pairs of adult monozygotic (13 pairs) than dizygotic (11 pairs) twins; sample sizes were too small to calculate heritability in this study [23].

Table 1. Population mean (Mean) and population variance (Standard deviation, SD) for Percent BOLD Signal Change (Ambiguous Facial expressions vs. Control Stimuli) and the Degree of Functional Connectivity (number of regions correlated with an r>0.3).

Region of Interest	Percent BOLD Signal Change		Degree of Functional Connectivity	
	Mean	Variance	Mean	Variance
L MVLFC	0.35	0.65	9.91	4.65
R MVLFC	0.43	0.63	10.20	4.87
L MDLFC	0.40	0.56	10.72	4.87
**R MDLFC**	**0.56**	**0.58**	**11.43**	**4.95**
**L PMC**	**0.37**	**0.66**	**10.46**	**5.07**
**R PMC**	**0.44**	**0.59**	**11.83**	**4.98**
**R PreSMA**	**0.37**	**0.72**	**7.88**	**5.06**
L Rhinal Sulcus	0.17	0.40	5.48	3.92
R RhinalSulcus	0.23	0.38	6.17	4.23
L Amygdala	0.37	0.63	7.36	4.44
R Amygdala	0.50	0.61	7.99	4.78
L Ant STS	0.27	0.67	8.02	4.88
**R Ant STS**	**0.44**	**0.52**	**10.45**	**5.19**
L Post STS	0.52	0.53	12.70	4.78
**R Post STS**	**0.73**	**0.54**	**14.03**	**4.44**
L FFA	0.54	0.62	11.60	4.66
R FFA	0.64	0.75	12.20	4.54
L LOC	0.47	0.70	12.29	4.27
R LOC	0.58	0.68	12.21	4.23
**L V2V3**	**0.37**	**0.58**	**9.99**	**4.65**
R V2V3	0.37	0.58	9.81	4.64
L Cerebellum	0.45	0.53	11.22	4.74
**R Cerebellum**	**0.22**	**0.52**	**8.89**	**4.67**
L Putamen	0.25	0.58	7.64	4.81
**R Putamen**	**0.33**	**0.60**	**8.42**	**4.99**

Regions in bold are those with GREML-based estimates of heritability of the brain response to ambiguous facial expressions (% BOLD Signal Change) significant at an alpha 0.05 (uncorrected).
Mid-ventrolateral frontal cortex (MVLFC); Mid-dorsolateral frontal cortex (MDLFC); premotor cortex (PMC), pre supplementary motor area (PreSMA); superior temporal sulcus (STS); fusiform face area (FFA); lateral occipital cortex (LOC); left (L); right (R).
doi:10.1371/journal.pgen.1004523.t001

In the ambiguous viewing condition, we observed that brain regions with the most population variance in their contribution to the face-processing network, as indexed by the "nodal degree" metric, were the most heritable. In other words, the amount of population variance in functional connectivity of a given region was related to the probability of explaining this region's response (to faces) by global genetic variations. Furthermore, we also observed that – across the 25 ROIs - brain regions with highest estimates of heritability (and highest population variance in connectivity) appear to coincide with ROIs characterized by intermediate values of their response to facial expressions (and their connectivity). As illustrated in Figure 4, the combination of these two properties (i.e., population mean and variance in nodal degree) sets apart two "subnetworks" we term "Obligatory" and "Optional" to denote their hypothetical role in processing faces. The proposed model builds on the distinction between the "Core" and "Extended" neural systems for face perception, as outlined originally by Haxby and colleagues [10]. In their model, the "Core" system consists of the inferior occipital gyri, fusiform gyri and a set of cortical areas along the superior temporal sulcus; this system includes all "Obligatory" regions identified here. The "Extended" system encompasses the anterior temporal cortex (e.g., face identity) and amygdala (emotion), as well as the auditory cortex (speech-related mouth movements) and cortical areas in the intra-parietal sulcus (spatial attention); with the exception of the anterior temporal cortex, this system is different from the "Optional" system consisting primarily of fronto-cortical regions. Nonetheless, both the "Extended" system of Haxby and colleagues and the Optional one identified here are viewed as being "…comprised of regions from neural systems for other cognitive functions that can be recruited to act in concert with the regions in the core system to extract meaning from faces" [10].

We suggest that the "Obligatory" regions are brought online in most participants when viewing human facial expressions; hence the high mean number of connections but low inter-individual variance in connectivity. These regions coincide with visual areas located in the occipital and temporal cortex, known to respond robustly to complex visual stimuli in general and human faces in particular. On the other hand, the "Optional" regions are co-opted to the face network by some but not all participants (hence the high population variance in their connectivity). Given the putative role of these regions in different aspects of working

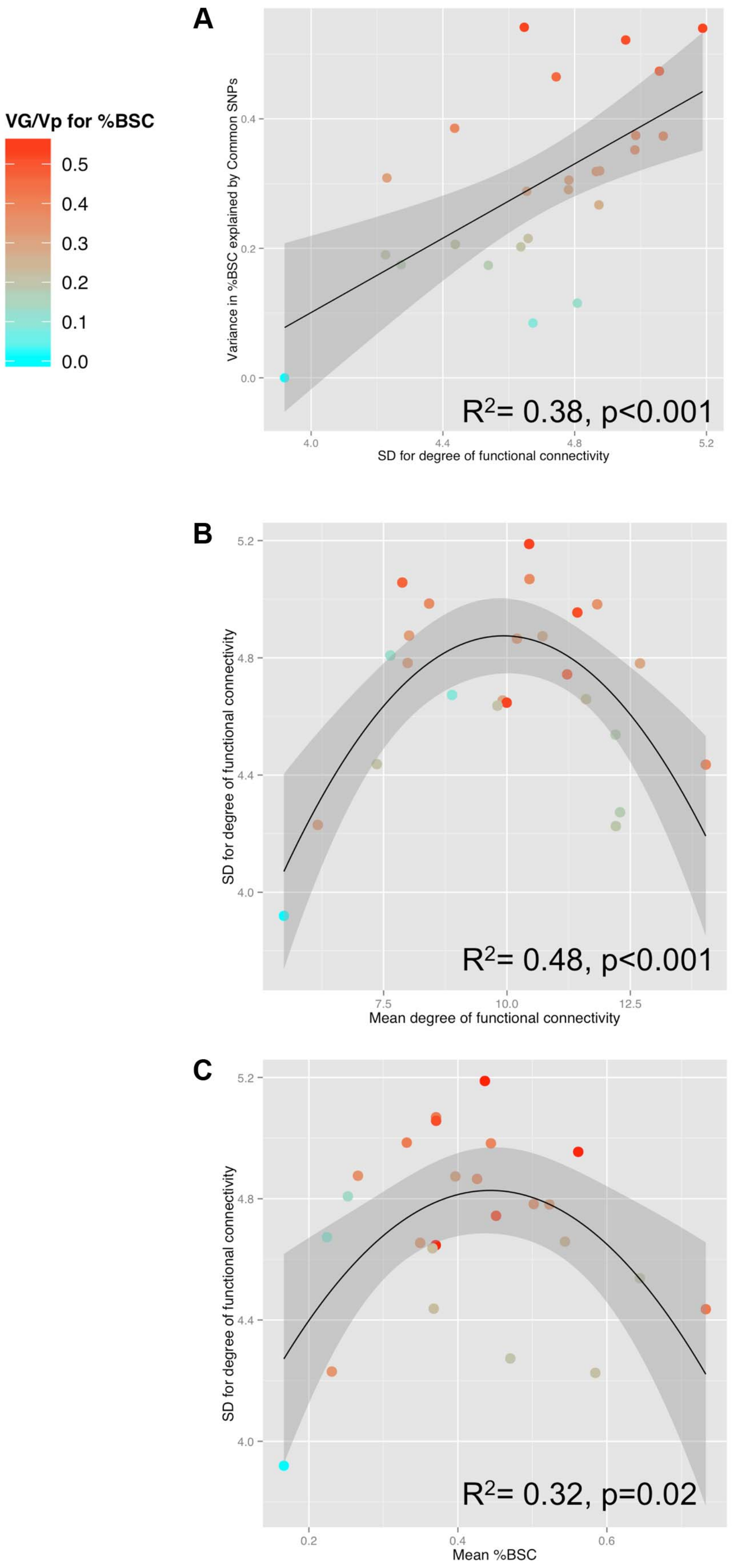
VG/Vp for %BSC
0.5
0.4
0.3
0.2
0.1
0.0
A
Variance in %BSC explained by Common SNPs
SD for degree of functional connectivity
R2= 0.38, p<0.001
B
SD for degree of functional connectivity
Mean degree of functional connectivity
R2= 0.48, p<0.001
C
SD for degree of functional connectivity
Mean %BSC
R2= 0.32, p=0.02

Figure 3. Relationships between SNP-based estimates of heritability, population variance in functional connectivity and the BOLD response. A) Relationship between GREML-based heritability estimates (Genetic Variance/Phenotypic Variance) and the population variance (standard deviation, SD) of functional connectivity (node degree) across 25 ROIs for the Ambiguous face viewing contrast. B) Relationship between the population variance (SD) and the population mean of degree across the 25 ROIs. C) Relationship between population mean of the brain response (percent BOLD signal change, %BSC) and the population variance (SD) of degree across the 25 ROIs. For all three plots, colour is scaled according to the GREML results for %BSC (cyan for low values and red for high values). VG, Genetic Variance; Vp, Phenotypic Variance.
doi:10.1371/journal.pgen.1004523.g003

memory (mid-dorsolateral and mid-ventrolateral prefrontal cortex) and motor resonance (premotor cortex), we speculate that individuals co-opting these regions while viewing faces do so in the context of their cognitive evaluation engaged spontaneously (no instructions were given to this effect). It is of interest to note that a number of "Optional" regions show shared genetic covariance with each other (albeit at marginal significance levels); this is not the case for any pair of "Obligatory" regions.

Finally, it appears that such a recruitment of the "Optional" regions may related to their connectivity with the posterior part of the STS (region #5 in Fig. 4), the only "Obligatory" region with a high number of connections to all four "Optional" regions. Of course, the posterior STS is an ideal candidate for bridging these two subnetworks, given its well-established role in extracting and processing social signals in non-biological motion [24]. This view is supported by our observation that the posterior STS is the only "Obligatory" region that shows genetic covariance with a number of "Optional" regions.

Overall, it is possible that regions with either low (floor effect) or high (ceiling effect) engagement by the stimulus (dynamic facial expressions) do not provide large enough range of (phenotypic) values across individuals and, in turn, are less likely to capture co-variations with genetic variance. Note that given the modest sample size (1,620 unrelated individuals), we are powered here to detect only relatively high values of heritability [25]. Recent studies suggest that more than half of narrow-sense heritability (h^2), as estimated through studies of related individuals, can be explained by common genetic variations assessed with SNPs included in most DNA microarrays [26]. Furthermore, the degree of GREML-based estimates of heritability predicts strongly the number of SNPs that reach nominal significance in a GWAS across 47 quantitative traits [15]. We have observed similar relationship for the brain response to faces across the 25 brain regions.

The low GREML-based estimates of heritability in the Angry condition were unexpected but clear cut: VG/Vp values were close to 0 in 19/25 ROIs, with values in the remaining ROIs varying between 0.12 and 0.28 (Supplemental Table S2). As explained in the "Limitations and Significance" section, we have power to detect only relatively high values of "heritability", albeit with fairly large confidence intervals. If true heritability of the brain response to Angry Facial expressions is low (this is unknown), we are underpowered to estimate such low values using the GREML-approach in the current study. An alternative possibility is that, in general, neural processing of angry facial expressions is as heritable as that of any other facial expressions but our paradigm fails to elicit an "adequate" brain response. Given our observation of the strong relationship between GREML-based estimates of heritability (VG/Vp) for Ambiguous Facial expressions and the population variance in functional connectivity (Figure 2), it is important to note that the latter is lower in the Angry (vs. Ambiguous) contrast. Furthermore, unlike the Ambiguous contrast (Figure 3c), there is no relationship between the mean BOLD response and the population variance in functional

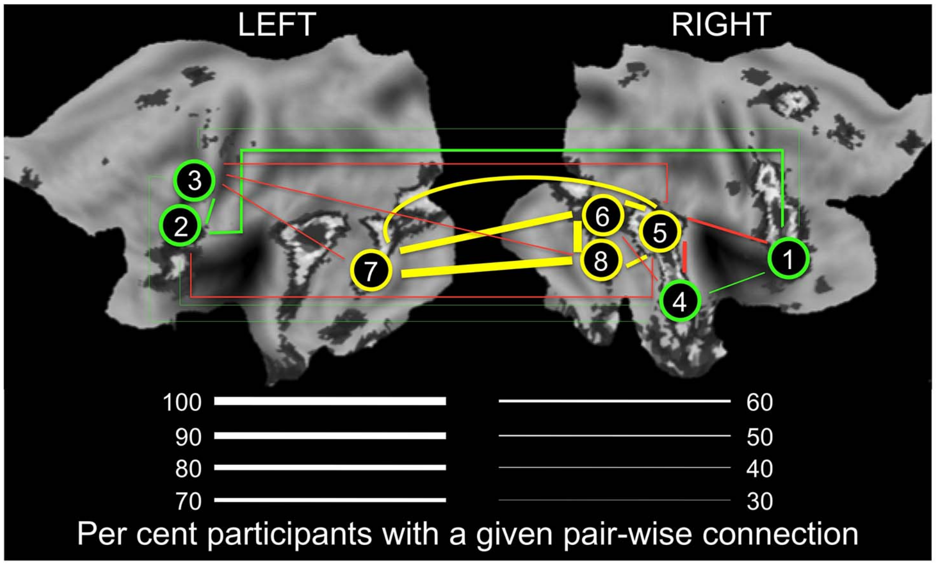

Figure 4. Connectivity in the "Obligatory" (yellow) and "Optional" (green) nodes of the face network. Thickness of lines indicates proportion of participants (%) with a given pair-wise connection, defined as a pair-wise correlation r>0.3. Yellow and green lines denote connections within the "Obligatory" and "Optional" networks, respectively. Red lines denote connections across the two subnetworks; for clarity, only connections present in 50% or more participants are shown. For all pair-wise values, see Supplementary Table S2. 1, mid-ventrolateral frontal cortex (right); 2, mid-dorsolateral frontal cortex (left); 3, premotor cortex (left); 4, anterior portion of the superior temporal sulcus (right); 5, posterior portion of the superior temporal sulcus (right); 6, fusiform face area (right); 7, lateral occipital cortex (left); 8, lateral occipital cortex (right). LEFT, the left hemisphere; RIGHT, the right hemisphere. The flat maps of the cerebral cortex contain the probability map of the face network adapted from Tahmasebi et al. [13].
doi:10.1371/journal.pgen.1004523.g004

Table 2. Proportion of individuals with connections between a given pair of regions of interest.

	MVLFCR	MDLFCL	PMCL	AntSTSR	PostSTSR	FFAR	LOCL	LOCR
MVLFCR	1.00	0.56	0.30	0.54	0.61	0.28	0.30	0.27
MDLFCL	0.56	1.00	0.60	0.35	0.45	0.35	0.31	0.32
PMCL	0.30	0.60	1.00	0.31	0.49	0.45	0.48	0.51
AntSTSR	0.54	0.35	0.31	1.00	0.65	0.47	0.37	0.37
PostSTSR	0.61	0.45	0.49	0.65	1.00	0.78	0.69	0.75
FFAR	0.28	0.35	0.45	0.47	0.78	1.00	0.85	0.89
LOCL	0.30	0.31	0.48	0.37	0.69	0.85	1.00	0.95
LOCR	0.27	0.32	0.51	0.37	0.75	0.89	0.95	1.00

Mid-ventrolateral frontal cortex (MVLFC); Mid-dorsolateral frontal cortex (MDLFC); premotor cortex (PMC); anterior superior temporal sulcus (AntSTS); posterior superior temporal sulcus (PostSTS); fusiform face area (FFA); lateral occipital cortex (LOC); left (L); right (R). The first four regions (MVLFCR, MDLFCL, PMCL, AntSTSR) constitute the "Optional" network while the other four regions (PostSTSR, FFAR, LOCL, LOCR) constitute the "Obligatory" network.
doi:10.1371/journal.pgen.1004523.t002

connectivity across the 25 ROIs in the Angry contrast (Supplemental Figure S2c). Thus, it is possible that angry facial expressions did not engage the face network in the same manner as the ambiguous facial expressions, resulting in a suboptimal phenotype.

Limitations and Significance

The key limitation of the present report is sample size; with 1,620 unrelated individuals, we are at the lower limit of the GREML-based approach for estimating contributions of common SNPs to phenotypic variations. Therefore, we were able to detect only relatively high values of heritability; note that these estimates have fairly large confidence intervals (standard error [SE]; e.g., R-MDLFC: VG/Vp = 0.52±0.22) and must be therefore interpreted cautiously. Simulations conditional on empirical GWAS data are consistent with these observations; sample size of 1,999 individuals is adequate for estimating correctly high ($h^2 = 0.5$) narrow-sense heritability [25]. The limited sample size also affected the significance values. Nonetheless, the uneven distribution of the nominally significant results between the Ambiguous (9/25) and Angry (0/25) speaks against a chance nature of these findings, as confirmed by the Monte-Carlo simulations.

The above sample-size limitation must be viewed in the context of the phenotype under study, however. The previous twin-based studies of fMRI-based phenotypes employed between 20 and 141 twin pairs, with heritability (a^2) estimates varying widely between 0 and 65 [27]. A total of 333 related individuals were included in a pedigree-based study of heritability of resting-state fMRI; h^2 for functional connectivity of the different components of so-called default-mode network varied between 10 (SE = 13) and 42 (SE = 17) [28]. Working with unrelated individuals, only a few other studies are acquiring functional brain phenotypes with a sample size comparable to the present report. For example, the Human Connectome Project plans to collect paradigm-based and resting-state fMRI datasets in 1,200 individuals [29]. In the Generation R cohort, scanning is under way to collected resting-state fMRI in up to 5,000 children (White, personal communication). Given the challenges related to test-retest reliability of fMRI data in general, and resting-state fMRI in particular, we suggest that the GREML approach provides an excellent test-bed for evaluating various approaches aimed at improving the fidelity of functional brain phenotypes. Such a GREML-based approach would be particularly powerful for fine-tuning functional phenotypes for meta-analyses of genome-wide association studies (similar to those carried out with structural brain phenotypes [30]), which require pooling of fMRI datasets (paradigm-based or resting) collected under varied conditions and on different scanners; GREML-based estimates of "heritability" would provide a useful metric for selecting appropriate post-processing steps and/or modifying inclusion criteria before launching the GWAS.

Overall, this report indicates that GREML-based estimates of heritability of the brain response to facial expressions vary across regions and paradigms, possibly as a function of inter-regional differences in the population variance of functional connectivity. As such, it demonstrates the usefulness of this approach in identifying functional phenotypes with properties suitable for genetic studies.

Materials and Methods

Participants

As part of the IMAGEN project [12], 2,000 adolescents (~14 years of age) were recruited through local high schools in eight European cities across four countries: France (Paris), Germany

(Mannheim, Hamburg, Dresden and Berlin), Ireland (Dublin) and United Kingdom (London and Nottingham). Local ethics boards approved the study protocol: Comité de protection des personnes Ile de France (CPP IDF VII); Ethics Committee of the German Psychological Society (DPG); Hamburg Chamber of Physicians Ethics Board (Hamburg Medical Association); Medical Ethics Commission of the Faculty of Clinical Medicine Mannheim; Medical Faculty Carl Gustav Carus Ethics Commission, Technical University Dresden; Nottingham University Medical School Research Ethics Committee; Psychiatry, Nursing & Midwifery Research Ethics Committee, King's College London; Ruprecht-Karls-University of Heidelberg; and School of Psychology Ethics Committee, Trinity College Dublin. The parents and adolescents provided written informed consent and assent, respectively.

MRI Acquisition and Initial Quality Control

Scanning was performed on 3 Tesla scanners from four different manufacturers (Siemens: 4 sites, Philips: 2 sites, General Electric: 1 site, and Bruker: 1 site). High-resolution T1-weighted anatomical images were acquired using 3D Magnetization Prepared Rapid Acquisition Gradient Echo (MPRAGE) sequence (TR = 2,300 ms; TE = 2.8 ms; flip angle = 9°; voxel size: $1.1 \times 1.1 \times 1.1$ mm^3). Functional T2*-weighted images were acquired using Gradient-Echo Echo-Planar-Imaging (GE-EPI) sequences (field of view: 22 cm; pixel size: 3.4×3.4 mm^2; slice thickness of 2.4 mm; slice gap 1.0 mm; effective final voxel size $3.4 \times 3.4 \times 3.4$ mm^3; TE = 30 ms and TR = 2,200 ms; flip angle = 75°).

During the fMRI session participants viewed short videoclips displaying ambiguous facial expressions (gestures such as nose twitching), angry facial expression or control stimuli (non-biological motion). The control stimuli were adapted from a study of Beauchamp and colleagues [31]. The face stimuli were created as follows. Eight actors (four females) were filmed for the face movements. They were instructed to express different emotions starting from a neutral point. We also extracted short video-clips from the periods when the actors were not expressing the emotions but were nonetheless moving their face (e.g. twitching their nose, opening their mouth, blinking their eyes). Twenty video-clips were selected for the angry and ambiguous face movements respectively. Four raters judged the intensity of each of emotion from those clips. The average rating for the angry face movements, on a scale of 1 (not angry at all) to 9 (very angry) was 7.94 (Standard Deviation [SD] = 0.77). The average rating for the ambiguous facial expressions was 2.18 (SD = 0.84) for anger, 2.97 (SD = 1.07) for sadness and 3.49 (S = 1.03) for happiness; combined across the three scales, the rating of ambiguous facial expressions was 2.92 (SD = 1.18). The control stimuli consisted of black-and-white concentric circles of various contrasts, expanding and contracting at various speeds, roughly matching the contrast and motion characteristics of the faces and hands clips [32]. We presented dynamic video clips of faces because, compared with static faces, they elicit more robust responses in brain regions critical for face processing, such as the fusiform gyrus and amygdala, and engage a more elaborate network for face processing, including regions in the frontal cortex and along the superior temporal sulcus [33]. The three viewing conditions were organized into 18-second blocks (5 Ambiguous, 5 Angry, 9 control) for a total of 160 EPI volumes in a single six-min fMRI run.

From 2,000 participants, a total of 1,926 completed both the Face paradigm and T1-weighted scan. Data from 79 participants were excluded due to excessive head movement during functional MR scanning (more than 2 *mm* in translation or 2 degrees in rotation errors in either direction), 8 participants were excluded due to unknown age, 5 participants had poor quality of fMRI data, and 1 participant was excluded because of abnormal ventricles. Scans from 1,831 participants were preprocessed using SPM8 toolbox (Statistical Parameter Mapping: Wellcome Department of Cognitive Neurology, London, UK) in MATLAB 7.0 (www.mathworks.com). Functional (EPI) images were motion-corrected with respect to the first volume. Subsequently, the EPI images were aligned to the corresponding high-resolution T1-weighted images (co-registration). The co-registered EPI images were transformed to the ICBM152 template space using the deformation parameters from the nonlinear registration of the corresponding structural image to the ICBM152 template. The nonlinear registration was achieved using the Unified Segmentation tool in SPM package. Further details of the pre-processing pipeline is provided in Tahmasebi et al. [13].

Face Network: Definition and Analysis

Regions of interest (ROIs) relevant for face processing were defined from a probabilistic map computed in a subsample (n = 1,110) of the IMAGEN dataset, as reported in Tahmasebi et al [13]. From this map, 25 ROIs were defined that are consistently (population probability >0.5) engaged during the ambiguous and angry face processing, relative to control (non-biological motion) condition. For each ROI, mean percent BOLD signal change (%BSC) for each ROI was extracted for all participants, as in Tahmasebi et al. [13], and analyzed as phenotypes of interest in GREML analyses. Values of %BSC were standardized (Z-Scored) for each acquisition site to account for scanner effects. Sex was added as a covariate.

The connectivity matrix for each face condition was calculated as follows. Nuisance covariates including white matter (WM) signals, and cerebrospinal fluid (CSF) signals were regressed out from the BOLD signals; WM and CSF voxels were identified by thresholding (at 90%) the WM and CSF tissue probabilistic maps from the ICBM152 standard template. For each ROI, the mean BOLD signal time-series was calculated by averaging the BOLD signal from all voxels constituting the ROI at every time point (160 time points in total). The BOLD time-series for each face condition were then realized by concatenating the mean-centered signal from the corresponding blocks (5 blocks per face condition and 9 blocks for control; each block consists of 8 time points), shifted by 2 TRs (4.4 s) to accommodate for the rise in the hemodynamic response. The correlation matrix was calculated between the time-series from every pair of the 25 ROIs. This yielded a 25-by-25 symmetric functional-connectivity matrix for each participant and face condition. We reduce these matrices into undirected graphs by thresholding each pair-wise correlation at $r > 0.3$. This creates a graph (network) with ROI's as nodes and edges between them representing functional connections. Within each participant, we calculate node degree for each ROI (node) to summarize the graph. Node degree is simply the count of other ROI's in which the BOLD time series correlates ($r>0.3$) with the given ROI. This analysis was performed with the Brain Connectivity Toolbox [17].

Given the importance of measurement error in estimating heritability, we have evaluated reproducibility of the brain response to facial expressions by correlating – across the 25 ROIs - the %BSC values obtained in two randomly selected subsamples: Group A (434 males, 483 females) and Group B (448 males, 459 females). In the absence of test-retest reliability measurements, such a cross-group comparison provides an indirect index of measurement error. As shown in Supplemental Figure S3, variations of the %BSC across the 25 ROIs were highly predictable in Group B from measures obtained in Group A ($R^2 = 0.96$).

Genotyping, Genomic-Relationship-Matrix REstricted Maximum Likelihood (GREML) and Genome-Wide Association Study (GWAS) Analyses

Whole genome data were acquired from 2,089 participants using Illumina Human610-Quad Beadchip and Illumina Human660-Quad Beadchip. Quality control of the genotypes was accomplished using Plink software [34]. Of the 588,875 SNPs overlapping present on both chips, a total of 42,506 single nucleotide polymorphisms (SNPs) were excluded for missingness of more than 5%, 15 individuals excluded for low genotyping rate (less than 97%), 16,385 SNPs were excluded for failing to reach Hardy-Weinberg equilibrium ($p <= 0.0001$), and 20,131 SNPs were excluded for low minor allele frequency ($MAF < 0.01$).

In total, 511,089 SNPs were used to calculate genetic relationship matrices using GCTA (http://www.complextraitgenomics.com/software/gcta). We excluded adolescents with a genetic relationship >0.05 (i.e., more related than 2nd degree cousins) to remove the influence of potential shared environment effects or familial causal variants not captured by SNPs. We included the top 10 principal components of the identity-by-state matrix as a covariate in all analyses to control for population stratification in our cohort. For each ROI, we have calculated GREML-based estimates of "heritability", defines as Genetic Variance [VG]/Phenotypic Variance [Vp], for the brain response to facial expressions (%BSC).

In order to examine the relationship between the VG/Vp estimates and the number of SNPs reaching a nominal level of significance [15] across the 25 ROIs, we have carried out Genome-wide Association Studies (GWAS) of %BSC using the same set of 1,620 unrelated adolescents. To do so, we used PLINK software [34]. Mean %BSC values where standardized (Z-Scores) in order to control for effects of Sex and Scanning Site. The top 10 principal components of the identity-by-state matrix as a covariate in all analyses to control for population stratification in our cohort.

Supporting Information

Figure S1 Phenotypic (% BOLD Signal Change) correlation matrices for the Ambiguous and Angry contrasts. Mid-ventrolateral frontal cortex (MVLFC); Mid-dorsolateral frontal cortex (MDLFC); premotor cortex (PMC), pre supplementary motor area (PreSMA); superior temporal sulcus (STS); fusiform face area (FFA); lateral occipital cortex (LOC); left (L); right (R).
(PDF)

Figure S2 A) Relationship between GREML-based heritability estimates (Genetic Variance/Phenotypic Variance) and the population variance (standard deviation, SD) of functional connectivity (node degree) across 25 ROIs for the Angry face viewing contrast. B) Relationship between the population variance (SD) and the population mean of degree across the 25 ROIs. C) Relationship between population mean of the brain response (percent BOLD signal change, %BSC) and the population variance (SD) of degree across the 25 ROIs. For all three plots, colour is scaled according to the GREML results for %BSC (cyan for low values and red for high values). VG, Genetic Variance; Vp, Phenotypic Variance.
(PDF)

Figure S3 Brain response (% BOLD Signal Change) response across the 25 ROIs measured in Group A and B. Left, males (Group A, n = 434; Group B, n = 448); Right, females (Group A, n = 483; Group B, n = 459).
(PDF)

Table S1 GREML results for percent BOLD Signal Change (%BSC) in response to Ambiguous Facial expressions (vs. Control Stimuli) in 1,620 adolescents. Regions in bold are those with GREML-based estimates of heritability of the brain response significant at an alpha 0.05 (uncorrected). The critical value for $X^2(1)$ in this context is 2.7055. Mid-ventrolateral frontal cortex (MVLFC); Mid-dorsolateral frontal cortex (MDLFC); premotor cortex (PMC), pre supplementary motor area (PreSMA); superior temporal sulcus (STS); fusiform face area (FFA); lateral occipital cortex (LOC); left (L); right (R). VG, Genetic Variance; Vp, Phenotypic Variance; df, degrees of freedom.
(DOC)

Table S2 GREML results for percent BOLD signal change (%BSC) in response to Angry Facial expressions (vs. Control Stimuli) in 1,620 adolescents. The critical value for $X^2(1)$ in this context is 2.7055. Mid-ventrolateral frontal cortex (MVLFC); Mid-dorsolateral frontal cortex (MDLFC); premotor cortex (PMC), pre supplementary motor area (PreSMA); superior temporal sulcus (STS); fusiform face area (FFA); lateral occipital cortex (LOC); left (L); right (R). VG, Genetic Variance; Vp, Phenotypic Variance; df, degrees of freedom.
(DOC)

Table S3 Population mean (Mean) and population variance (Standard deviation, SD) for Percent BOLD Signal Change (Angry Faces vs. Control Stimuli) and the Degree of Functional Connectivity (number of regions correlated with an $r > 0.3$). Mid-ventrolateral frontal cortex (MVLFC); Mid-dorsolateral frontal cortex (MDLFC); premotor cortex (PMC), pre supplementary motor area (PreSMA); superior temporal sulcus (STS); fusiform face area (FFA); lateral occipital cortex (LOC); left (L); right (R).
(DOC)

Table S4 Results of bivariate analysis of genetic covariances in percent BOLD Signal Change (%BSC) in response to Ambiguous Facial expressions (vs. Control Stimuli) in 1,620 adolescents across regions of interest (ROI) constituting the "Optional" MVLFCR, MDLFCL, PMCL, AntSTSR) and "Obligatory" (PostSTSR, FFAR, LOCL, LOCR) Networks. Regions in bold and underlined are those with GREML-based estimates of genetic covariance (rG) of the brain response in a given pair of ROIs significant at an alpha 0.05 and 0.1 (uncorrected). Standard errors of the estimates are in parentheses. Mid-ventrolateral frontal cortex (MVLFC); Mid-dorsolateral frontal cortex (MDLFC); premotor cortex (PMC), superior temporal sulcus (STS); fusiform face area (FFA); lateral occipital cortex (LOC); left (L); right (R); Ant, Anterior; Post, posterior.
(DOC)

Acknowledgments

We thank Nick Qiu and Klara Mareckova for their assistance with processing and quality control of the MR images. We thank three anonymous reviewers for their insightful comments that allowed us to improve this report.

Members of the IMAGEN consortium (www.imagen-europe.com)

Institute of Psychiatry, King's College, London, United Kingdom

G. Schumann, P. Conrod, L. Reed, G. Barker, S. Williams, E. Loth, A. Lourdusamy, S. Costafreda, A. Cattrell, C. Nymberg, L. Topper, L. Smith, S. Havatzias, K. Stueber, C. Mallik, T-K. Clarke, D. Stacey, C. Peng Wong, H. Werts, C. Andrew, S. Desrivieres, F. Carvalho.

Department of Psychiatry and Psychotherapy, Campus Charité Mitte, Charité – Universitätsmedizin Berlin

A. Heinz, J. Gallinat, I. Häke, N. Ivanov, A. Klär, J. Reuter, C. Palafox, C. Hohmann, C. Schilling, K. Lüdemann, A. Romanowski, A. Ströhle, E. Wolff, M. Rapp

Physikalisch-Technische Bundesanstalt, Berlin, Germany
B. Ittermann, R. Brühl, A. Ihlenfeld, B. Walaszek, F. Schubert
Institute of Neuroscience, Trinity College, Dublin, Ireland
H. Garavan, C. Connolly, J. Jones, E. Lalor, E. McCabe, A. Ní Shiothcháin, R. Whelan.
Department of Psychopharmacology, Central Institute of Mental Health, Mannheim, Germany
R. Spanagel, F. Leonardi-Essmann, W. Sommer
Department of Cognitive and Clinical Neuroscience, Central Institute of Mental Health, Mannheim
H. Flor, F. Nees, M. Struve
Department of Child and Adolescent Psychiatry, Central Institute of Mental Health, Mannheim, Germany
T. Banaschewski, L. Poustka, S. Steiner
Department of Addictive Behaviour and Addiction Medicine
K. Mann, M. Buehler, S. Vollstaedt-Klein
Department of Genetic Epidemiology in Psychiatry, Central Institute of Mental Health, Mannheim, Germany
M. Rietschel, E. Stolzenburg, C. Schmal
Schools of Psychology, Physics and Biomedical Sciences, University of Nottingham, United Kingdom
T. Paus, P. Gowland, N. Heym, C. Lawrence, C. Newman, Z. Pausova
Technische Universitaet Dresden, Germany
M. Smolka, T. Huebner, S. Ripke, E. Mennigen, K. Muller, V. Ziesch
Department of Systems Neuroscience, University Medical Center Hamburg-Eppendorf, Hamburg, Germany
C. Büchel, U. Bromberg, T. Fadai, L. Lueken, J. Yacubian, J. Finsterbusch
Institut National de la Santé et de la Recherche Médicale, Service Hospitalier Frédéric Joliot, Orsay, France
J-L. Martinot, E. Artiges, F. Gollier Briand, J. Massicotte, N. Bordas, R. Miranda, Z. Bricaud, Marie L. Paillère Martinot, N. Pionne-Dax, M. Zilbovicius, N. Boddaert, A. Cachia, J-F. Mangin
Neurospin, Commissariat à l'Energie Atomique, Paris, France
J-B. Poline, A. Barbot, Y. Schwartz, C. Lalanne, V. Frouin, B. Thyreau
Behavioural and Clinical Neurosciences Institute, Department of Experimental Psychology, University of Cambridge, United Kingdom
J. Dalley, A. Mar, T. Robbins, N. Subramaniam, D. Theobald, N. Richmond, M. de Rover, A Molander, E Jordan

Author Contributions

Conceived and designed the experiments: TB GJB AB CB PC HF HG JG PG AH BI CL KM JLM FN EL ZP MR MNS AS RT GS TP. Analyzed the data: EWD AT LF. Contributed reagents/materials/analysis tools: NK ML. Wrote the paper: EWD TP ZP GJB LF TN.

References

1. Bourke C, Douglas K, Porter R (2010) Processing of facial emotion expression in major depression: a review. Aust N Z J Psychiatry 44: 681–696. doi:10.3109/00048674.2010.496359.
2. Machado-de-Sousa JP, Arrais KC, Alves NT, Chagas MHN, de Meneses-Gaya C, et al. (2010) Facial affect processing in social anxiety: tasks and stimuli. J Neurosci Methods 193: 1–6. doi:10.1016/j.jneumeth.2010.08.013.
3. Marwick K, Hall J (2008) Social cognition in schizophrenia: a review of face processing. Br Med Bull 88: 43–58. doi:10.1093/bmb/ldn035.
4. Toh WL (2011) Current visual scanpath research: a review of investigations into the psychotic, anxiety, and mood disorders. Compr Psychiatry 52: 567–579.
5. Leppänen JM (2006) Emotional information processing in mood disorders: a review of behavioral and neuroimaging findings. Curr Opin Psychiatry 19: 34–39. doi:10.1097/01.yco.0000191500.46411.00.
6. Blair RJR (2010) Neuroimaging of psychopathy and antisocial behavior: a targeted review. Curr Psychiatry Rep 12: 76–82. doi:10.1007/s11920-009-0086-x.
7. Harms MB, Martin A, Wallace GL (2010) Facial emotion recognition in autism spectrum disorders: a review of behavioral and neuroimaging studies. Neuropsychol Rev 20: 290–322. doi:10.1007/s11065-010-9138-6.
8. Taylor SF, MacDonald AW 3rd, Cognitive Neuroscience Treatment Research to Improve Cognition in Schizophrenia (2012) Brain mapping biomarkers of socio-emotional processing in schizophrenia. Schizophr Bull 38: 73–80. doi:10.1093/schbul/sbr105.
9. Zhu Q, Song Y, Hu S, Li X, Tian M, et al. (2010) Heritability of the specific cognitive ability of face perception. Curr Biol CB 20: 137–142. doi:10.1016/j.cub.2009.11.067.
10. Haxby Hoffman, Gobbini (2000) The distributed human neural system for face perception. Trends Cogn Sci 4: 223–233.
11. Yang J, Lee SH, Goddard ME, Visscher PM (2011) GCTA: a tool for genome-wide complex trait analysis. Am J Hum Genet 88: 76–82. doi:10.1016/j.ajhg.2010.11.011.
12. Schumann G, Loth E, Banaschewski T, Barbot A, Barker G, et al. (2010) The IMAGEN study: reinforcement-related behaviour in normal brain function and psychopathology. Mol Psychiatry 15: 1128–1139. doi:10.1038/mp.2010.4.
13. Tahmasebi AM, Artiges E, Banaschewski T, Barker GJ, Bruehl R, et al. (2012) Creating probabilistic maps of the face network in the adolescent brain: a multicentre functional MRI study. Hum Brain Mapp 33: 938–957. doi:10.1002/hbm.21261.
14. Robert CP CG (2005) Monte Carlo Statistical Methods. 2nd ed. Springer.
15. Yang J, Lee T, Kim J, Cho M-C, Han B-G, et al. (2013) Ubiquitous polygenicity of human complex traits: genome-wide analysis of 49 traits in Koreans. PLoS Genet 9: e1003355. doi:10.1371/journal.pgen.1003355.
16. Deary IJ, Yang J, Davies G, Harris SE, Tenesa A, et al. (2012) Genetic contributions to stability and change in intelligence from childhood to old age. Nature 482: 212–215. doi:10.1038/nature10781.
17. Rubinov M, Sporns O (2010) Complex network measures of brain connectivity: uses and interpretations. NeuroImage 52: 1059–1069. doi:10.1016/j.neuroimage.2009.10.003.
18. Lee SH, DeCandia TR, Ripke S, Yang J, Sullivan PF, et al. (2012) Estimating the proportion of variation in susceptibility to schizophrenia captured by common SNPs. Nat Genet 44: 247–250. doi:10.1038/ng.1108.
19. Grueter M, Grueter T, Bell V, Horst J, Laskowski W, et al. (2007) Hereditary prosopagnosia: the first case series. Cortex J Devoted Study Nerv Syst Behav 43: 734–749.
20. Wilmer JB, Germine L, Chabris CF, Chatterjee G, Williams M, et al. (2010) Human face recognition ability is specific and highly heritable. Proc Natl Acad Sci 107: 5238–5241. doi:10.1073/pnas.0913053107.
21. Greenwood TA, Braff DL, Light GA, et al (2007) Initial heritability analyses of endophenotypic measures for schizophrenia: The consortium on the genetics of schizophrenia. Arch Gen Psychiatry 64: 1242–1250. doi:10.1001/archpsyc.64.11.1242.
22. Anokhin AP, Golosheykin S, Heath AC (2010) Heritability of individual differences in cortical processing of facial affect. Behav Genet 40: 178–185. doi:10.1007/s10519-010-9337-1.
23. Polk TA, Park J, Smith MR, Park DC (2007) Nature versus nurture in ventral visual cortex: a functional magnetic resonance imaging study of twins. J Neurosci Off J Soc Neurosci 27: 13921–13925. doi:10.1523/JNEUROSCI.4001-07.2007.
24. Allison, Puce, McCarthy(2000) Social perception from visual cues: role of the STS region. Trends Cogn Sci 4: 267–278.
25. Zaitlen N, Kraft P (2012) Heritability in the genome-wide association era. Hum Genet 131: 1655–1664. doi:10.1007/s00439-012-1199-6.
26. Zaitlen N, Kraft P, Patterson N, Pasaniuc B, Bhatia G, et al. (2013) Using extended genealogy to estimate components of heritability for 23 quantitative and dichotomous traits. PLoS Genet 9: e1003520. doi:10.1371/journal.pgen.1003520.
27. Blokland GAM (2012) Genetic and Environmental Influences on Neuroimaging Phenotypes: A Meta-Analytical Perspective on Twin Imaging Studies. Twin Res Hum Genet 15: 351–371.
28. Glahn DC, Winkler AM, Kochunov P, Almasy L, Duggirala R, et al. (2010) Genetic control over the resting brain. Proc Natl Acad Sci U A 107: 1223–1228. doi:10.1073/pnas.0909969107.
29. Van Essen DC, Smith SM, Barch DM, Behrens TE, Yacoub E, et al. (2013) The WU-Minn Human Connectome Project: an overview. Neuroimage 80: 62–79. doi:10.1016/j.neuroimage.2013.05.041.
30. Stein JL, Medland SE, Vasquez AA, Hibar DP, Senstad RE, et al. (2012) Identification of common variants associated with human hippocampal and intracranial volumes. Nat Genet 44: 552–561. doi:10.1038/ng.2250.
31. Beauchamp MS, Lee KE, Haxby JV, Martin A (2003) fMRI responses to video and point-light displays of moving humans and manipulable objects. J Cogn Neurosci 15: 991–1001.
32. Grosbras M-H, Paus T (2006) Brain Networks Involved in Viewing Angry Hands or Faces. Cereb Cortex 16: 1087–1096. doi:10.1093/cercor/bhj050.
33. Fox CJ (2009) Defining the face processing network: Optimization of the functional localizer in fMRI. Hum Brain Mapp 30: 1637–1651.
34. Purcell S, Neale B, Todd-Brown K, Thomas L, Ferreira MAR, et al. (2007) PLINK: a tool set for whole-genome association and population-based linkage analyses. Am J Hum Genet 81: 559–575. doi:10.1086/519795.
